# An Analysis of Soil Coring Strategies to Estimate Root Depth in Maize (*Zea mays*) and Common Bean (*Phaseolus vulgaris*)

**DOI:** 10.34133/2020/3252703

**Published:** 2020-11-08

**Authors:** James D. Burridge, Christopher K. Black, Eric A. Nord, Johannes A. Postma, Jagdeep S. Sidhu, Larry M. York, Jonathan P. Lynch

**Affiliations:** ^1^The Pennsylvania State University, Department of Plant Science, Tyson Building, University Park, PA 16802, USA; ^2^Department of Biology, Greenville University, 315 E. College Ave, Greenville, IL 62246, USA; ^3^Forschungszentrum Jülich GmbH, Institute of Bio-and Geosciences-Plant Sciences (IBG-2), 52425 Jülich, Germany; ^4^Noble Research Institute, LLC, 2510 Sam Noble Parkway, Ardmore, OK 73401, USA

## Abstract

A soil coring protocol was developed to cooptimize the estimation of root length distribution (RLD) by depth and detection of functionally important variation in root system architecture (RSA) of maize and bean. The functional-structural model *OpenSimRoot* was used to perform *in silico* soil coring at six locations on three different maize and bean RSA phenotypes. Results were compared to two seasons of field soil coring and one trench. Two one-sided *T*-test (TOST) analysis of *in silico* data suggests a between-row location 5 cm from plant base (location 3), best estimates whole-plot RLD/D of deep, intermediate, and shallow RSA phenotypes, for both maize and bean. Quadratic discriminant analysis indicates location 3 has ~70% categorization accuracy for bean, while an in-row location next to the plant base (location 6) has ~85% categorization accuracy in maize. Analysis of field data suggests the more representative sampling locations vary by year and species. *In silico* and field studies suggest location 3 is most robust, although variation is significant among seasons, among replications within a field season, and among field soil coring, trench, and simulations. We propose that the characterization of the RLD profile as a dynamic rhizo canopy effectively describes how the RLD profile arises from interactions among an individual plant, its neighbors, and the pedosphere.

## 1. Introduction

The spatiotemporal deployment of roots in soil is of increasing interest as agricultural scientists seek to understand how root placement relates to the capture of soil resources [1–7] and carbon sequestration [8, 9]. Optimizing soil resource acquisition while limiting resource loss and increasing carbon sequestration would contribute needed resilience to a world experiencing climate change and soil degradation [2, 10–12]. Recent research has supported the connection between root deployment and resource acquisition in a variety of crops, see Tracy et al. [13] and Ye et al. [7] for recent general reviews. Substantial opportunity costs and tradeoffs mean that simply increasing overall root length is not the best strategy for all environments [2].

Therefore, it is important to effectively characterize root length distribution across depth in annual row crops. Root length within a given soil layer is commonly reported as root length density (RLD) with units cm root per cm^3^ soil [4, 14]. There is much debate on protocol selection and specifically on how to balance intensive and extensive sampling [15, 16]. Protocol selection depends on research goals, resource availability, genotypic and environmental variation at a particular site, and balancing the likelihood of errors due to under-sampling with eventual diminishing returns of additional sampling [9]. Field research is a critical component of this effort and *in situ* methods employed include monolith excavation, trench excavation, augers or soil coring, and minirhizotrons [17].

Monolith excavation involves the excavation of large blocks of soil, often in a three-dimensional gridded pattern, over a large area from which roots are typically removed by washing [18–20]. Monoliths provide rich data but are very time consuming and destructive, hence, few samples are generally obtained, which means variation across a field and through time can be masked. Trench excavation typically uses manual shoveling or mechanical excavators to expose a wall of soil on which a grid can be applied so roots can be counted or traced [21]. A trench is useful for combining observations of soil characteristics as well as the RLD profile but is also time-consuming, destructive, and does not facilitate sampling an extensive area. Other techniques such as the pinboard technique [22], root impact on plane method [23, 24], and root mapping on three perpendicular planes [25, 26] have been effectively used to gauge relative root distribution but are difficult to translate to root length and have many of the same advantages and disadvantages as soil coring. Correlation among coring locations as well as among methods of estimating the RLD profile is not always apparent [27] nor is it clear which method indicates functionally important phenotypic differences [28]. Hecht et al. [29] address this challenge by estimating nodal root angle in barley with two coring locations using a relatively simple algorithm involving D_50_, the depth at which 50% of root length is found. Miguel et al. [30] used *in silico* soil coring and were able to better distinguish shallow and deep-rooted root architectural phenotypes 15 cm from the plant row, as compared to 5 cm from the plant row.

Soil coring is commonly used to quantify root distribution because a greater number of samples can be taken over an extensive area. A variety of methodologies have been proposed with different size cores [31–34], numbers, and locations of cores [17, 35], often with decisions based upon throughput or convenience rather than empirical research. Oikeh et al. [36] follows Wiesler and Horst [34] in taking multiple subsamples per plot, using a single core location, relative to focal plants, and combining subsamples. Böhm [15] recommends 5 cores per plot. Several authors have observed significant differences among coring locations in a plot [37–40]. Bengough et al. [17] calculated the number of replicates needed to differentiate means 22% different from each other with 40% CV and indicate 25 replicates would be needed to have a 50% chance of differentiating means. Morandage et al. [41] studied variation among techniques using virtual soil coring, minirhizotrons, and trenching of maize and wheat RSA and found 10-50 cores would be needed to obtain a 10% relative standard error but the question remains as to how well virtual models represent actual variation in the RLD profile.

Multiple groups have attempted to use monoliths as a “ground truth” and then used this data to simulate soil coring in various ways. Gajri et al. [42] compared seven coring locations against four monoliths in maize (30 cm perpendicular to row ×22.5 cm parallel to row ×10 cm deep) and recommend a single core location with two to three samples and four replicates, approximately 10 cm from the plant in-row. Buczko et al. [43] excavated one monolith composed of 10 cm cubes over an area measuring 70 cm (perpendicular to plant rows) by 40 cm (parallel to plant rows) by 30 cm (deep) and compared hypothetical core locations to the monolith data set. With some reservation, they recommend taking two cores per plot, one in-row and one between-row, and applying a 1 : 3 weighting scheme, or taking at least eight cores per plot [43]. Wu et al. [44] performed monolith excavations of maize root systems, 3D mapped the washed root system, and then performed simulated soil coring based upon the excavated root system. They generated an algorithm correcting RLD for coring location involving one or preferably two cores per plot. A limitation of these monolith-based approaches is that the low number of samples may not capture a representative amount of variation.

Noninvasive techniques such as magnetic resonance imaging (MRI), X-ray computed tomography (CT), and positron emission tomography (PET) (for a brief description see [45]) offer nondestructive time-series observations [46–49] describing the three-dimensional structure of root architecture. However, these methods cannot be used reliably in the field, and measurements are constrained to relatively small pots, which introduce artifacts [50]. Other technologies such as ground-penetrating radar (GPR) are being used in forestry [51] and have agronomic applications to the coarser roots of perennial crops such as fruits and tuber crops such as cassava [52, 53]. Detection of naturally occurring, or added tracers such as oxygen isotopes, heavy water, or deuterium offer estimates of the depth of resource acquisition [54–56].

The variety of adjustments and algorithms suggested by the previous work are based on the particular environments studied. Application of these algorithms to other environments can be further limited by differences in core location and row and plant spacing. An extensive comparison of coring locations was performed by Ordóñez et al. [57], who were interested in determining a plot-level RLD profile. They compared 4 coring locations and developed a simple weighted average algorithm that accurately predicts the RLD profile by adjusting for distance from row. In the end, they suggest the coring location most, the representative of the plot level average is 10 cm from the plant row, similarly to other work [32, 36, 42]. Other work by Ordóñez et al. [58] maps the root front velocity and maximum depth of maize and soybean using two core locations per plot.

It is generally accepted that overly simplistic sampling is prone to error [22, 35, 42], that observed variance in RLD decreases as sample number increases [33, 34, 59] and that results can be affected by core location [60]. Identifying an optimum coring strategy is important and difficult [17]. Complications include environmental variability, genotype by environment interactions, the inherently plastic nature of root growth, and the artifacts of any particular protocol. Designing an efficient sampling protocol requires recognizing that total variation is composed of (1) systematic variation related to coring location and root architecture and (2) random variation related to the dynamic interactions among the individual, its community, and the pedosphere. Holding systematic factors constant, even if it produces a known bias in RLD estimates, may permit better characterization of random variation. In this study, we compared an RLD estimation algorithm using Voronoi shapes, which are irregular polyhedrons defined by the relative proximity of one point to another, to an unadjusted RLD estimate. We tested both methods on field studies and used simulated soil coring of phenotypes with contrasting RLD profiles to identify the optimum method to estimate whole plot root length, to identify optimum coring locations for estimating root length, and to identify optimum coring locations for detecting differences between root architectural phenotypes.

## 2. Methods

### 2.1. Simulation

**Figure 1:**
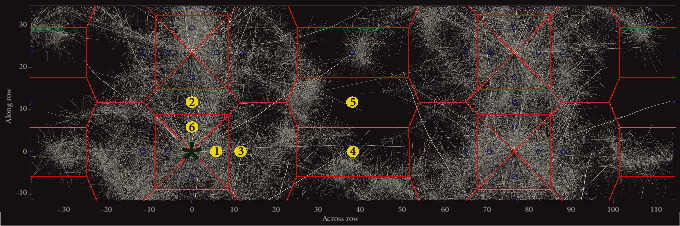
Top view of coring locations with four maize plants. Yellow circles represent core locations, identified with respect to the focal plant (green asterisk). Blue circles represent possible alternate placements of coring locations by rotation and/or reflection. The red polygons are the Voronoi map showing the area estimated by each coring location. Row spacing for both species was 76 cm, in row spacing for bean was 10 cm and 23 cm for maize. Between row bean coring locations (1, 3, 4) were oriented identically but in row positions (2, 5, 6) were compressed due to tighter in row planting density of bean.

Maize and bean root system architectures with three contrasting root angles were simulated using the functional-structural model *OpenSimRoot* [61]. Each simulation consisted of a single plot containing four maize or four bean plants grown for 40 days under nonlimiting conditions. The parameter sets for the three simulated phenotypes of each crop differed only in axial or basal root branching angle, which was altered for every root class by incrementing *OpenSimRoot's* defaults by 0°, +20°, or+40°, respectively, for shallow, intermediate, and deep bean, and by -30°, 0°, and +30° for maize. Row spacing was 76 cm for both species while in-row spacing was 10 cm for bean and 23 cm for maize. The acquisition of 44 mm diameter soil cores, which matches the core diameter used in the field, at 40 days after planting was simulated by extracting RLD in 10 cm depth increments at the 6 locations depicted in Figure 1 and at a random location that varied in every simulation. Root length from the entire plot by depth was also extracted in 10 cm depth increments and divided by soil volume to obtain the whole-plot root length distribution by depth (RLD) profile. Each phenotype was simulated 100 times, with variation between replicates provided by specifying gravitropism, root extension rate, branching frequency, and root tip deflection as stochastic variables. Full parameterizations and model outputs are available at Zenodo 10.5281/zenodo.3952179.

### 2.2. Field Experiment

Maize (*Zea mays*) inbred line Mo17 and common bean (*Phaseolus vulgaris*) variety Windbreaker were grown on a sandy loam soil at the Apache Root Biology Center (Wilcox, AZ 32.03, -109.69). Soil at this site is a mix of fluvial and lacustrine sediments from an ancient lakebed (Lake Cochise) and is classified as a Grabe loam [62]. Agronomic conditions were nonlimiting, and plant growth was normal in both years. Bean was planted on July 3, 2015, and maize was planted on June 1, 2015. In 2016, planting was June 4 for bean and May 25 for maize. In both years, between-row spacing was 76 cm while in-row spacing was 10 cm for bean and 23 cm for maize.

Stainless steel soil cores (Giddings Equipment Company) with a 44 mm internal diameter and length of 60 cm were inserted into the soil using dead blow sledgehammers and removed by hand. Cores were taken July 17-18, 2015, and July 27-28, 2016, in five locations for bean, six locations for maize, and with eight replications for each species. The bean field coring procedure in 2016 omitted location 4 because the tighter planting density of bean, as compared to maize, and limitations to perfectly align seed placement across rows obviated the difference between locations 4 and 5 in bean. A depiction of core locations is presented in Figure 1. Each core was taken from a different fully bordered and healthy plant. Each replication was taken from a different 5m × 5m area. Cores were stored at 4°C until divided into 10 cm segments and gently washed by hand using low-pressure water over a 2 mm screen. Roots were stored in 25% alcohol until imaged using an Epson V700 flatbed scanner at 300 dpi and analyzed using WinRhizo Pro (Regent Instruments).

On July 26, 2016, 52 days after planting of bean and 62 days after planting for maize, an excavator was used to dig a trench spanning 5 rows to a depth of 160 cm. The face of the wall was cut smooth using a hand shovel, and all roots protruding from the face were cut flush with the wall. Surveyor string was used to create a grid, with each cell measuring 14 cm (x) by 7.6 cm (y). Observations of soil texture and structure were taken for each grid cell. Soil texture was classified into categories of loose loam, blocky clay peds, or dense but penetrable sandy loam. Soil structure was categorized as either relatively unstructured or as composed of blocky peds. Low-pressure water was used to gently wash three to five cm of soil from the trench face and expose roots. Exposed root were then cut from each cell, placed in 25% alcohol, stored at 4°C until scanned, and analyzed with WinRhizo as described above.

### 2.3. Statistical Analysis

To compare *in situ* and *in silico* RLD estimates derived from different core locations (or subsets thereof), we utilized a Voronoi diagram of the coring locations. This divides the plot into irregular polyhedra with dividing lines having equal proximity to adjacent core locations. Voronoi areas are an objective metric that by definition adjusts for differences in row and plant spacings. The RLD profile from individual coring locations was aggregated to estimate the whole-plot RLD profile by weighting coring locations based on the Voronoi areas associated with each coring location. This was based on two assumptions: (1) the coring locations could be reflected (flipped 180 degrees across the row and rotated 180 degrees around the focal plant), so a location 5 cm from the focal plant, in the row, could equivalently be on either side of the focal plant, (2) the RLD profile in the area nearer any coring location (its Voronoi area), or in the Voronoi area of any of the reflections or rotations of that location, was best represented by that coring location. Voronoi-adjusted RLD refers to the whole-plot RLD calculated using the weighted mean of the *n* coring locations, with weights representing the relative sizes of the Voronoi areas associated with each coring location.

(1) $$\begin{array}{c} {\text{RLD}}_{\text{adj}}=\overset{n}{\underset{i=1}{\sum }}\left({\text{RLD}}_{i}\times w_{i}\right), \end{array}$$

where *n* is the number of coring locations, RLD_*i*_ is the RLD of the *i*^th^ coring location, and *w*_*i*_ is the Voronoi-area derived weight for the *i*^th^ coring location. The unweighted mean of *n* coring locations was also calculated, since it is the typical approach, and is termed the unadjusted average. The reference values for RLD are either the Voronoi-adjusted RLD or the unadjusted average RLD for all six coring locations.

To confirm that the simulated phenotypes of each crop are different by depth or not, a two-way ANOVA was conducted on the whole-plot average. The model included the main effects of phenotype, depth, and interaction between phenotype and depth. ANOVA results were followed by Tukey honest significant differences (HSD) test by depth. The same ANOVA procedure was repeated using the Voronoi weighted average.

To determine which method of estimating the RLD profile best estimated true whole-plot RLD, we compared both the unadjusted mean of all six cores and the Voronoi-adjusted method against the whole-plot RLD using Two one-sided *T*-test (TOST) analysis, which is an equivalence test of whether an observation falls within defined bounds. Tests were conducted by phenotype and by depth among the actual whole-plot average, the unadjusted mean of the six core locations, and the Voronoi-adjusted mean of the 6 core locations. When subsets of the six coring locations are used to estimate the whole-plot RLD profile, Voronoi areas were calculated for only the core locations being used. Equivalence was declared if the *p* value was less than 0.05. An R script was developed to automate this data processing and includes the ability to calculate the Voronoi-adjusted RLD profile as well as the unadjusted RLD profile for all subsets of core locations. It is publicly available on Zenodo at 10.5281/zenodo.3952179.

The depth above which 50% and 95% of roots are found is a univariate metric used to gauge rooting depth. In this method, the cumulative sums of root length per soil core segment, from zero to sixty in ten-centimeter increments, are used to calculate the depth at which the give percentage of roots is reached [63]. It can also be calculated by combining core segments of the same depth from different locations or calculated based on the actual whole-plot RLD profile in the case of simulations.

To understand how sampling location affects the ability to detect phenotype differences in rooting depth, we performed a resampling analysis. For each simulated core, we computed univariate depth metrics (*D*_50_, *D*_80_, *D*_90_, *D*_95_; the depth above which 50%, 80%, etc. of roots were located). We then repeatedly sampled from our universe of model outputs to select sets of 2-20 simulated cores from each sampling location and recorded whether one-way ANOVA found significant differences (*p* < 0.05) in each depth metric among the three phenotypes. We performed 2000 replicates of this whole procedure for each sample size and considered the proportion of resamples producing significant ANOVAs to be an indicator of how reliably cores from that location would detect differences in root depth.

Multivariate quadratic discriminant analysis (QDA) (in R Package MASS) was used to identify a coring location(s) that best distinguished the *OpenSimRoot* generated root angle phenotypes (shallow, intermediate, and deep) for each crop. Equal proportion of all phenotypes was assumed. QDA was selected over linear discriminant analysis (LDA) because QDA allows for different variance structures among classes (phenotypes in this case) while LDA requires equal variance. QDA calculates quadratic score functions unique to each phenotype, which each has its own mean vectors and variance-covariance matrices. A QDA decision rule (equation below) is applied to classify new samples from the test set among the different phenotypes, and a new sample is assigned to a phenotype with the largest ${\hat{S}}_{i}^{Q}\left(x\right)$ score.

(2) $$\begin{array}{c} {\hat{S}}_{i}^{Q}\left(x\right)=-\frac{1}{2}\log\left|S_{i}\right|-\frac{1}{2}{\left(x-\bar{x_{i}}\right)}^{\prime }S_{i}^{-1}\left(x-\bar{x_{i}}\right)+\log p_{i}, \end{array}$$

where ${\hat{S}}_{i}^{Q}\left(x\right)$ is the quadratic discriminant function for *x* (sample) in phenotype *I*, *S*_*i*_ is covariance matrix of *i*^th^ phenotype, ${\bar{x}}_{i}$ is mean vector of *i*^th^ phenotype, *x* is a vector for each location by depth observation, and *p*_*i*_ is the probability of *i*^th^ phenotype occurring (0.5 if two phenotypes are to be classified but 0.33 if three phenotypes are to be classified).

From the 100 simulated root systems of each phenotype, 50 were randomly selected and reserved as a test set. Subsequently, multiple random training sets of varying sample sizes, i.e., 8, 10, 15, 20, 25, 30, 35, 40, 45, and 50 were selected. Using each of the training sets, the QDA decision rule was created for each of the coring locations, except for locations 4 and 5 (half-way between rows) because of rank deficiency for these two coring locations. Rank deficiency, or collinearity among multiple variables, was likely the result of recovering very few or no roots in the shallower soil zones at these between-row locations. The misclassification error rate and accuracy for each of the QDA sets were calculated using the test set and indicate the frequency at which a given phenotype is correctly categorized. This procedure was first performed to classify three phenotypes of each crop and then on only deep vs. shallow classification for each crop. Manhattan distance (sum of absolute differences in all depths) was used to compare RLD estimates from single or multiple coring locations to the reference values.

## 3. Results

### 3.1. Root Length Density Profile Estimation

**Figure 2:**
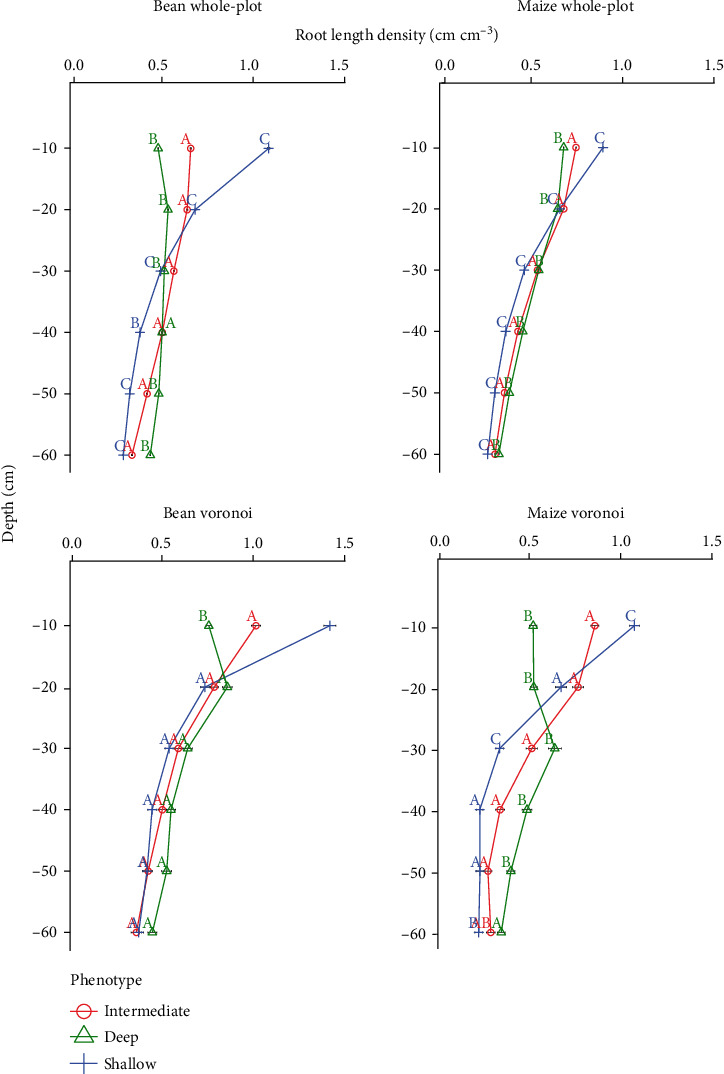
Root length density profile (RLD) of three simulated phenotypes for bean and maize using whole-plot average and the Voronoi-adjusted method. Lowercase letters indicate Tukey honest significant differences by depth at alpha level of 0.05 and horizontal bars represent standard error. One hundred cores were taken of each location by specie combination.

The three RSA phenotypes generated by *OpenSimRoot* differed in total root length by less than 3% (Supplementary Figure 1), but with contrasting depth distributions. In both maize and bean, RLDs of all three phenotypes were different from each other at every depth but one (Tukey HSD *p* < 0.05; Figure 2), with the sole exception that deep and intermediate bean were not distinguishable from each other in the 30-40 cm layer. ANOVA was also used to analyze the Voronoi-adjusted RLD profile for the three simulated root phenotypes, based upon the six coring locations. Significant differences in Voronoi-adjusted RLD were found among all three maize phenotypes in the 0-10 and 20-30 cm layers and between two maize phenotypes in the 10-20, 30-40, 40-50, and 50-60 cm layers but only at the 0-10 cm layer in bean (Figure 2).

**Figure 3:**
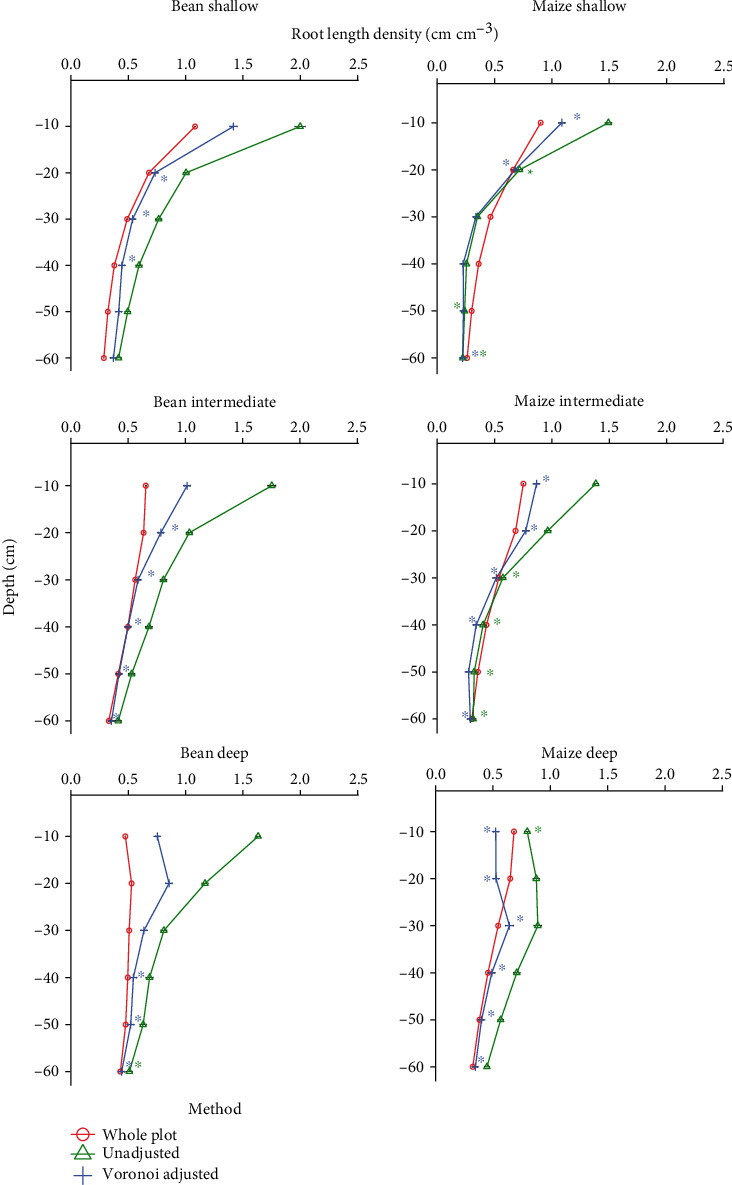
Root length density by depth for the three simulated phenotypes of bean and maize using the actual whole-plot average, unadjusted estimates, and Voronoi-adjusted estimates. Stars indicate TOST significant equivalence to whole-plot average, and horizontal bars represent standard error. One hundred cores were taken of each location by species combination.

To determine which method of estimating RLD from cores came closest to the true whole-plot value *in silico*, the unadjusted mean of all six cores were compared to against the Voronoi-adjusted method using a Two one-sided *T*-test (TOST) analysis for each phenotype. Both the unadjusted and Voronoi methods generally overestimated shallow RLD (Figure 3), but the Voronoi-adjusted method was not statistically different from the actual value in 25 of 36 cases, while the unadjusted method was not different from the actual value in only 8 of 36 cases. Deep phenotypes of both species showed the poorest agreement between estimated and actual RLD profiles.

**Figure 4:**
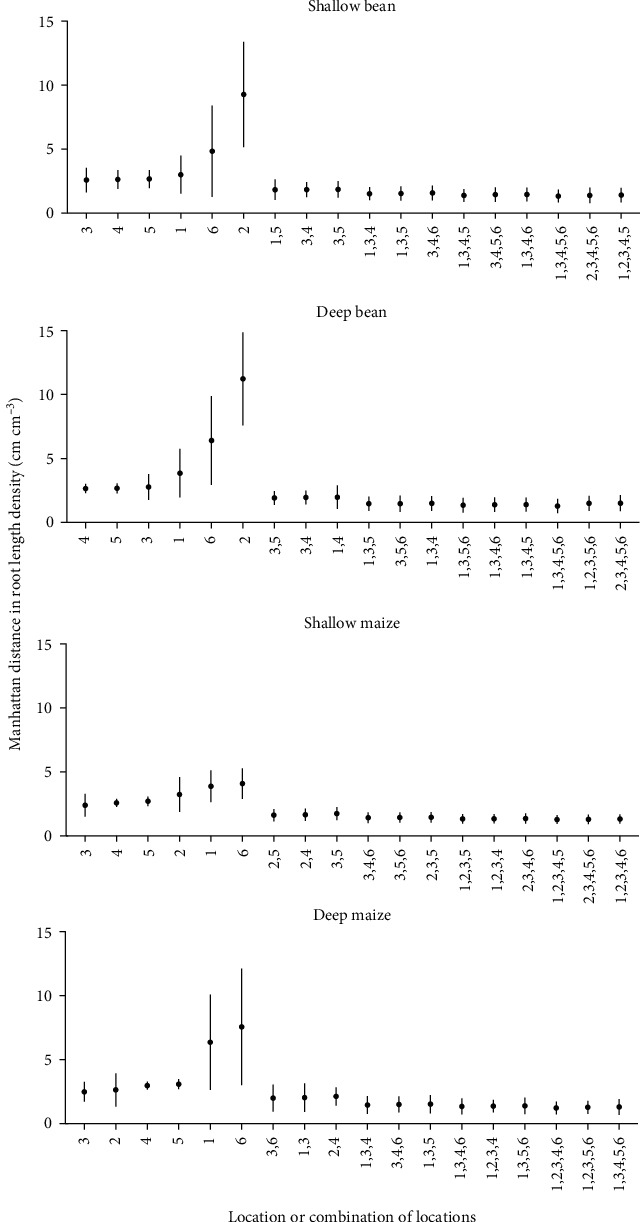
Manhattan distances between actual whole-plot RLD (cm cm^−3^) and estimated RLD (cm cm^−3^) from individual and combined soil coring locations for simulated shallow and deep RSA phenotypes. Dots represent the mean and bars represent the standard deviation. Only the best combinations of cores are displayed. The three combinations of coring locations with the lowest Manhattan distance are displayed for each category (combination of 2, 3, 4, and 5 core locations). One hundred replications of each location by species combination were taken.

**Figure 5:**
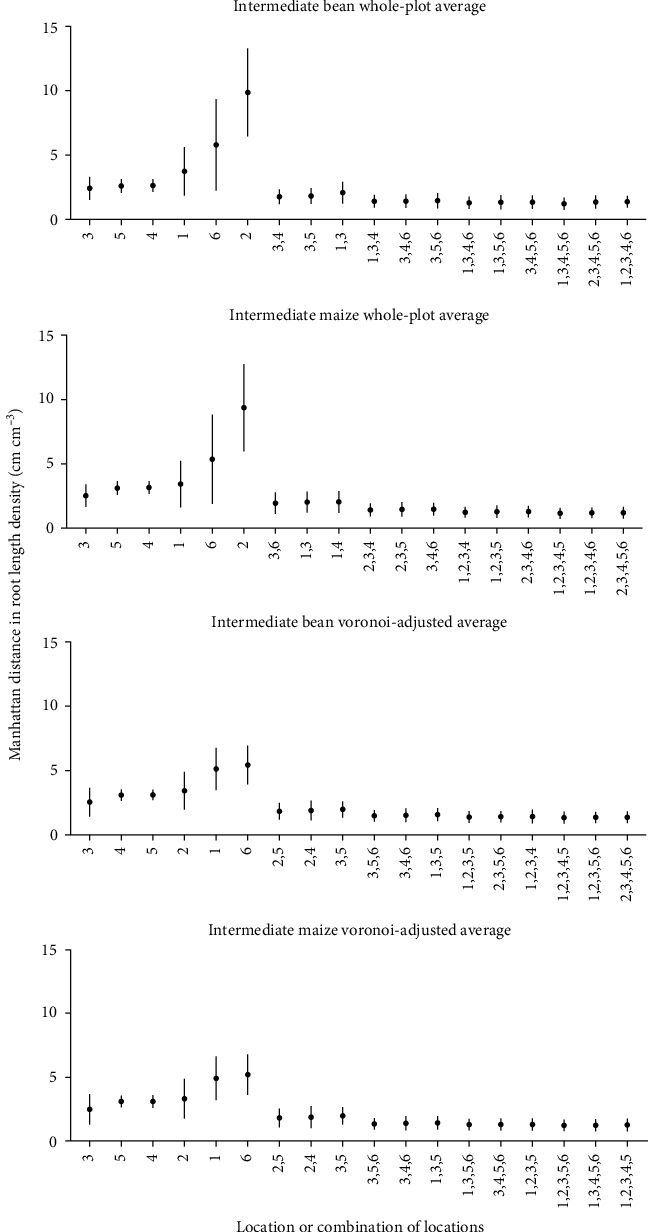
Manhattan distances between actual whole-plot RLD (cm cm^−3^) (top two panels) or Voronoi-adjusted RLD (cm cm^−3^) (bottom two panels) and estimated RLD (cm cm^−3^) from individual and combined soil coring locations for simulated intermediate RSA phenotypes. Dots represent mean and bars represent standard deviation. The three combinations of coring locations with the lowest Manhattan distance are displayed for each category (combination of 2, 3, 4, and 5 core locations). One hundred replications of each location by species combination were taken.

The whole-plot root length density (cm root cm^−3^ soil) (RLD) and Voronoi-adjusted whole-plot RLD of 100 replications of three simulated bean and maize phenotypes were compared to individual and combined coring locations. While location 3 (between row, 5 cm from plant base) was not always the best estimator of whole-plot RLD, it was a consistently good estimator across all phenotypes and species (Figures 4 and 5). Combinations of two coring locations reduce the difference between estimated RLD and actual RLD and reduce the variance of estimates, but the best combination varies by phenotype and species (Figures 4 and 5). Comparison of Voronoi-adjusted estimates of whole-plot RLD to individual and combined soil coring locations also highlights location 3 as a good estimator (Figure 5).

**Figure 6:**
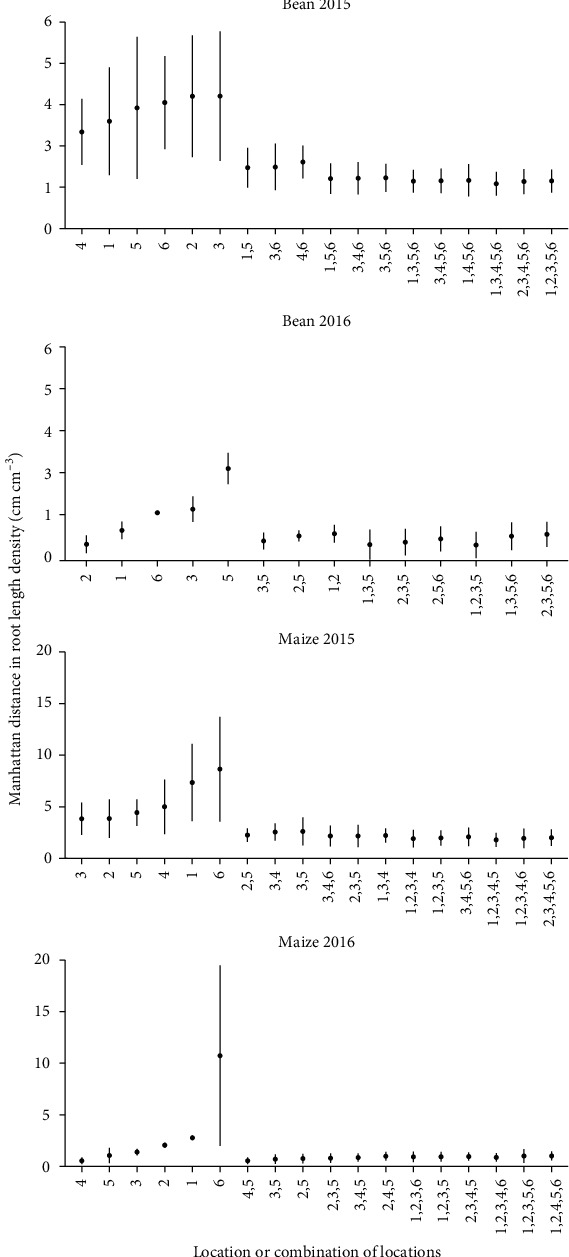
Manhattan distances between Voronoi-adjusted RLD (cm cm^−3^) and estimated RLD (cm cm^−3^) from individual and combined soil coring locations for 2015 and 2016 field trials. Dots represent mean and bars represent standard deviation. The three combinations of coring locations with the lowest Manhattan distance are displayed for each category (combination of 2, 3, 4, and 5 core locations). Seven replications of each location by species combination were taken.

**Figure 7:**
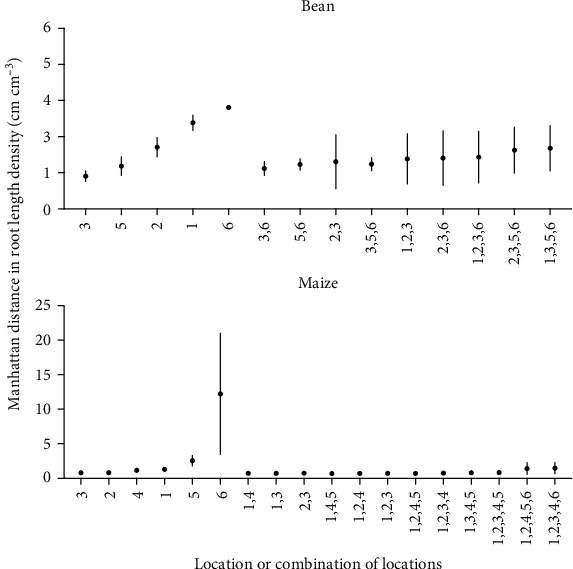
Manhattan distances between trench derived RLD (cm cm^−3^) and estimated RLD (cm cm^−3^) from individual and combined soil coring locations for the 2016 field trial. Dots represent mean and bars represent standard deviation. The three combinations of coring locations with the lowest Manhattan distance are displayed for each category (combination of 2, 3, 4, and 5 core locations). One hundred replications of each location by species combination were taken.

For the field sampling, estimated whole-plot RLD from the two seasons of soil coring and one season of trench excavation was compared to individual and combined soil coring location estimates using the Manhattan distances between actual and estimated RLD. For maize, location 3 was the best single estimator of Voronoi-adjusted whole-plot RLD in 2015 and 2016 as well as trench RLD (Figures 6 and 7). Statistical testing is limited by having only one trench per species. Combining locations improved estimates of whole-plot RLD but ideal core combinations were not consistent across seasons nor method (Voronoi-adjusted, trench). For bean, single locations 4 (halfway between plants in neighboring rows) and 1 (between row, next to plant base) were best in 2015, while in 2016, locations 2 (halfway between plants in a row) and 1 gave the better estimates (Figure 6). Combining locations reduced the Manhattan distance between estimates of whole-plot RLD, particularly when pairing a between-row location with an in-row location (Figure 6). Comparing estimates of RLD from individual core locations to the trench estimate of whole-plot RLD indicates mid-row locations 4 (halfway between plants in neighboring rows) and 5 (equidistant from 4 plants in 2 rows) were more accurate for bean, while locations 3 and 5 were more accurate for maize (Figure 7).

**Figure 8:**
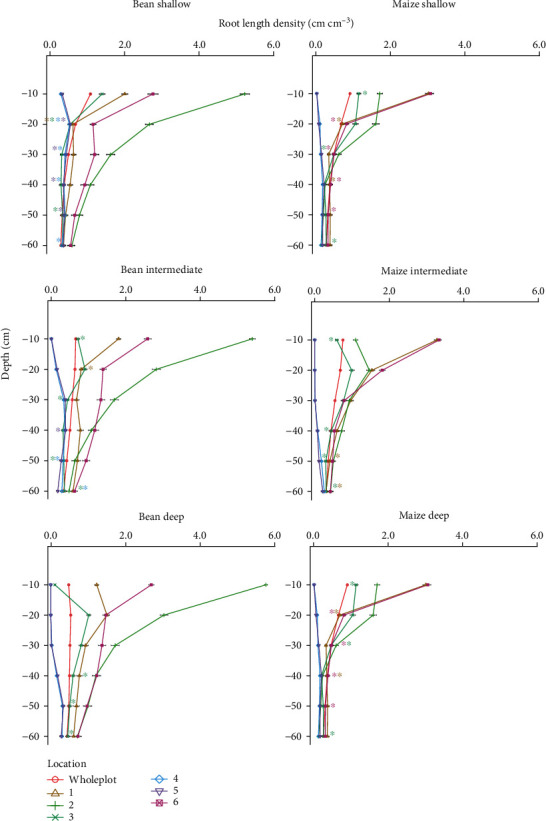
Root length density (cm cm^−3^) by depth profiles of the three simulated bean and maize root architectures obtained from 6 different soil coring locations and whole-plot average. Stars indicate significant equivalence to the whole-plot average using TOST analysis. Location is indicated by color and shape of dot, bars indicate standard error, and one hundred replications of each location by species combination were taken.

TOST analysis was used to identify the coring location that best estimates the whole-plot RLD profile and the Voronoi-adjusted RLD profile (Figure 8). As gauged by the number of times TOST analysis revealed significant similarities between estimation methods, location 3 was closest to the whole-plot RLD profile for the deep and intermediate phenotypes. The shallow maize phenotype was best estimated by using locations 6 (next to plant, in row) or 3, and the shallow bean phenotype was best estimated using locations 5 or 4. However, maize location 6 greatly overestimated shallow RLD, and bean locations 5 and 4 significantly underestimated shallow RLD.

### 3.2. Phenotypic Differentiation

When comparing univariate metrics of relative rooting depth, the largest differences between RSA phenotypes were seen at location 2 for maize (Supplementary Figures 2 and 3) and for bean at locations 4 and 5, followed by random core placement (Supplementary Figure 3). Differences between phenotypes were detected more reliably by *D*_50_ than by *D*_95_ at most coring locations (Supplementary Figure 3), possibly because the 60 cm coring depth imposed a limit on variation in observed *D*_95_. However, all depth indexes were able to differentiate maize RSAs in ~60% of cases with 5 replications at location 2, and *D*_50_ reached ~70% reliability with 5 replications at locations 1 and 6. Bean RSAs were differentiated by *D*_50_ in ~70% of cases at location 5 with five replications (Supplementary Figure 3), while at locations 1, 2, and 6, no depth metric was able to differentiate in more than ~30% of cases even with as many as 20 replications.

**Figure 9:**
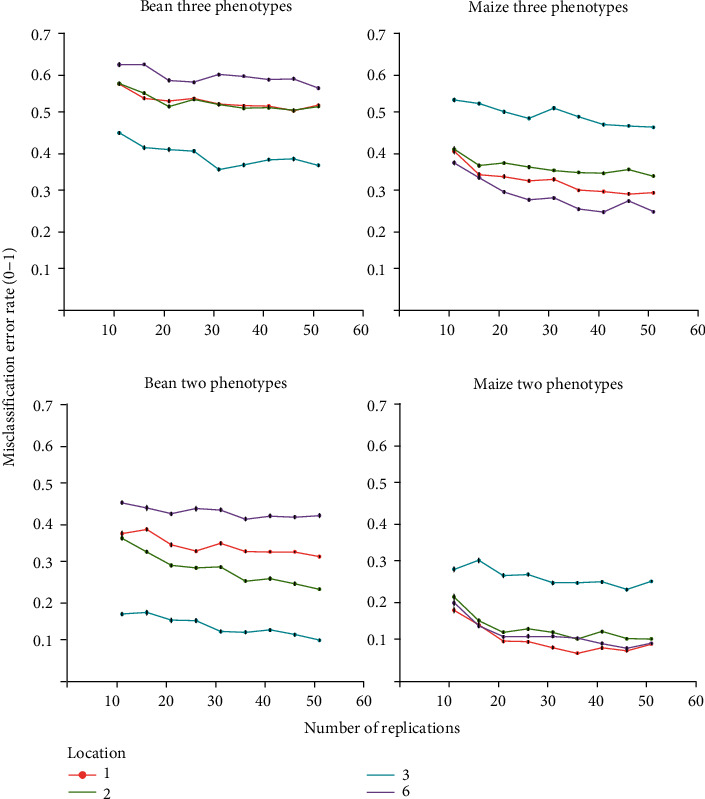
Visualization of quadratic discriminant analysis showing misclassification rate among three simulated bean and maize phenotypes and between two maize and bean phenotypes. Bars represent standard error.

The multivariate quadratic discriminant analysis supported the finding that some coring locations are better able to distinguish simulated phenotypes than others. For classifying three phenotypes, location 3 was the best for bean with ~70% accuracy, and location 6 was superior for maize with ~85% accuracy (Figure 9 and Table 1). Accuracy, equivalent to low misclassification error rate, improves if only shallow and deep phenotypes are considered, to ~95% at location 3 for bean and~95% at location 6 or 1 for maize (Figure 9 and Table 1). Pairs of coring locations were tested, and the best subset is presented in Table 1. Pairs of coring locations did not improve the misclassification error rate, defined as accuracy subtracted from the true value, between two common bean phenotypes but did slightly improve the misclassification error rate among three bean phenotypes from 0.36 to 0.34 (Table 1). The misclassification error rate among three maize phenotypes improved from 0.20 to 0.14 by adding another core, and from 0.05 to 0.02 between two maize phenotypes by adding another core.

### 3.3. Metrics of Variation

In order to translate observations based on simulations to the field, where it is impossible to efficiently quantify whole-plot root length density, we compared individual and paired core locations to the estimated whole-plot RLD profile using two techniques; the Voronoi-adjusted algorithm involving all six soil coring locations and by the trench-derived RLD profile. Metrics of variation among locations and among replicates were large *in silico* (Supplementary Figure 4) as well as in the field where we also observed large variation by year (Supplementary Figure 5). Some core locations were more prone to variation than others (Supplementary Figure 6). The RLD profile in 2016 was markedly different than in 2015 for both maize and bean and 2016 root length in the 10-20 zone was reduced by half and more than half at 0-10 cm (Supplementary Figure 6). Significant variation in the RLD profile was visible across the columns in the bean trench and seemed to be associated with a heterogeneously compacted zone.

**Figure 10:**
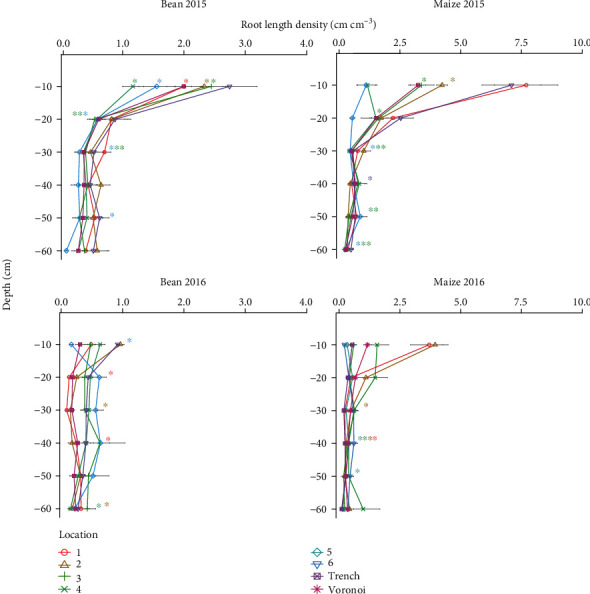
RLD by depth profiles obtained from field-based soil coring for bean 2015, bean 2016, maize 2015, and maize 2016. (c, d) include 2016 trench data. Stars indicate TOST equivalence to Voronoi-adjusted RLD profile, bars indicate standard error, and 7 cores were taken for each location by species combination.

We identified core location 3 as providing reasonably good estimates of the Voronoi-derived RLD profile in 2015 for both maize and bean, as gauged by the number of depths at which estimates were equivalent to the Voronoi average. However, in 2016 where less RL was recovered and data appears noisier, location 1 was better for bean, and locations 2 and 5 provided better estimates for maize (Figure 10). The Voronoi-adjusted average reported less RLD in shallow zones than did Voronoi estimates for maize, but for bean, the trench reported greater RLD in shallow zones than did Voronoi (Figure 10).

## 4. Discussion

Our goal was to balance intensive sampling requirements with limited resources and to cooptimize both detection of differences in whole-plot root length and RLD depth profile and categorization of functionally different RSA phenotypes. To this end, we used simulations to develop a soil coring protocol that requires as few cores as possible while enabling estimation of whole-plot RLD, RLD profile, and detection of whether RSA is shallow, intermediate, or deep. We were also able to determine that while *D*_50_ is better than *D*_95_ at differentiating phenotypes, quadratic discriminant analysis has the advantage of including the whole depth profile in a multivariate analysis.

### 4.1. Recommendation

Simulations suggest location 3 (between row, 5 cm from plant base) for both bean and maize best estimate whole-plot RLD (Figures 4 and 5) and that the whole-plot RLD profile is best estimated for the majority of phenotypes at location 3 (Figure 8). Soil core resampling and calculation of *D*_50_ and *D*_95_ of simulated deep, intermediate, and shallow phenotypes suggests *D*_50_ is a better metric than *D*_95_ for distinguishing RSA phenotypes (Supplementary Figure 6). This is likely due to larger phenotypic differences at *D*_50_ than *D*_95_ (Supplementary Figures 7 and 8). Quadratic discriminant analysis indicated location 3 for bean and location 6 (in row, next to plant base) for maize are best at being able to differentiate deep, intermediate, and shallow RSA (Figure 9). Quadratic discriminant analysis indicates 20 replications at location 3 for bean, and 10 replications at location 6 for maize, provide approximately 70% RSA phenotype categorization accuracy (Figure 9).

### 4.2. Variation Is Composed of Systemic and Random Factors

Because the plants simulated by *OpenSimRoot* were effectively invariant in overall root length (Supplementary Figure 1), all between-replicate variation in the root length seen at a given coring location (Supplementary Figure 2) was driven solely by fine-scale local variation in the rate, direction, and tortuosity of the growth of individual simulated root axes. Therefore, these simulations should be considered as a best-case scenario; as is clear from comparing the variance of simulated cores (Figure 6) to that of field data (Figure 8). These simulations did not account for additional real-world sources of variation such as heterogeneity in the growth environment, imprecision in core placement or depth partitioning, or variation in whole-plant growth rate.

While variation between field seasons is significant and results are variable, results indicate that location 1 (between row, next to plant) for bean and 3 (between row, 5 cm from plant base) for maize best approximate Voronoi-adjusted RLD (Figure 6). The closest RLD values to trench estimates for maize come from locations 3 or 5 (between row, 5 cm from plant base and halfway between rows, respectively) and location 4 or 5 (halfway between row locations) for bean (Figure 7). Field studies indicate pairs of core locations can improve estimates of RLD and the RLD profile (Figures 4–7). Simulations also show pairs of coring locations slightly improve misclassification error rates (Table 1).

The lack of congruity among these recommendations begs the question as to the source of the variation. We propose firstly that variation is real and worthy of study [17] and that a new perspective may be useful to reorient our approach. A rhizo canopy arises from the dynamic interactions among an individual plant, its neighbors, and the pedosphere, like a forest canopy expands dynamically in time and space. Even leaf area development and canopy closure of row crops, like soybean, is a complex process with incompletely understood effects of environment, neighbors, and management [64, 65]. Analogous attempts to characterize dynamic heterogeneity of tree branch structure [66], a forest canopy [67, 68], a city skyline [69], or traces of even historic human land use [70], have traditionally relied on transects, but technologies such as light detection and ranging (LiDAR) have dramatically increased coverage, resolution, and changed the way these processes are conceptualized ([71]; Anderson et al., 2006). As the applications of LiDAR are showing, a single small transect cannot be expected to capture all variation of a forest canopy or city skyline. Neither should a small number of trenches, monoliths, or cores be expected to capture all variation in a rhizo canopy. Regardless, variation in root distribution profile at the plant, plot, and field level may be an adaptive trait [72]. This variation may be better categorized as phenotypic plasticity ([73–75]: [76]) and flexibility, which should be considered on at least the whole organism level rather than the single trait level [77].

In spite of variation and its potential utility, we can however highlight that our field and simulation studies suggest location 3 is closest to whole-plot, Voronoi-adjusted and trench derived whole-plot RLD profile at the shallowest zone, which is an important zone for distinguishing phenotypes. We view this as the most important objective because this may enable breeders to identify functional differences in rooting depth. Although sampling at fixed times and locations can easily give a snapshot, albeit biased, of a spatiotemporally complex process, breeders interested in selecting for particular phenotypes often do not require absolute accuracy, but simple detection of relative differences (e.g., phenotype or environment).

In the context of crop breeding, differentiating phenotypes is more important than estimating whole-plot RLD (Figures 4 and 5) or estimating *D*_50_ or *D*_95_ phenotypes (Supplementary Figure 3). The between-row locations (4 and 5) may be good estimators of whole-plot RLD because locations closer to plants tend to overestimate whole-plot RLD. Furthermore, between-row locations underestimate shallow RLD (Figures 8 and 10, Supplementary Figures 7 and 8), which further reduces their value. However, accepting bias in comparative measurements does have hazards: it reduces the value of those measurements to future researchers and increases the chance of their being misinterpreted or reused after publication in ways that ignore the bias [78].

Contrasting results between maize and bean were expected due to fundamental differences in plant structure and planting density. Bean is a dicot with more higher-order laterals that does not usually generate new axial roots after 2-3 weeks from germination [79]. Maize is a monocot that generates successive whorls of nodal roots with increasing diameter through at least the silking stage [32]. Differences between maize and bean root initiation and secondary thickening result in ecologically significant contrasts in exploration strategies which may be involved in the different results and amount of variation between the species. The strategy of bean roots, that initially have small diameters but can then have secondary growth [80], may rely more heavily on the exploitation of pores, cracks, and fractures as does soybean [81], rather than penetration of mechanically impeded soil domains, as maize can [82]. Indeed, it is likely that roots can follow ped structure, fractures, or pores [83–85] to attain greater rooting depth in deep soil domains [86–91] where the strength required for roots to deform soil is outside their observed range [87]. These highly variable factors may be germane to explaining the heterogeneous and dynamic nature of root growth in the field.

Multiple studies investigating RLD have peripherally noted how compacted strata probably affected their results [32, 37]. Effects of localized impedance on root growth were evident in the bean trench. Impedance in columns P, Q, and R may be related to a relative proliferation of roots in columns M and N at the first and second depths, and column O at second and third depth (Supplementary Figure 9). It appeared that roots grew around the impeded zone, rather than through it, and then resumed a downward trajectory. While it is known that RLD variation can be large in a small plot and neighbors can have profound effects [92], the microvariation in the structure and texture of a soil has not been intensively studied. It is likely that local responses to microvariation can be complex, multidimensional, and nonadditive [93, 94]. Recognizing the diversity of soil conditions and genotype × environment responses, even in the same genotype and field trial, may lead to identifying biologically relevant trends or strategies [95].

In contrast to Orndóñez et al. [57] who found no differences in root length or root mass between two experimental locations and Liedgens and Richner [96] who found no difference between RLD across years, Hirte et al. [97], as well this research, found RLD to be substantially different in two years and among replications (Supplementary Figures 5 and 6). We speculate that this difference could be due to the variable soil structure, texture, and bulk density between the locations. Nevertheless, assuming that a uniform rooting front exists [58] or that all individuals in a plot are similar [43, 44, 98] overlooks potentially important variation among individual plants, locations in the field, or across more distinct soil types or agroecosystems. It is unclear if a particular coring location consistently provides good estimates of RLD, and the RLD profile across environments and phenotypes. Assumptions of homogenous root growth are even less likely to be accurate in more heterogeneous, nutrient-limited, or compacted soils [27, 34, 89, 90, 99]. Furthermore, root growth plasticity may be a biologically important trait and has been observed in response to soil drying [100–102], depth to water table [103], and nitrogen availability [104].

### 4.3. Conclusion

We used field and simulation studies to develop a soil coring protocol that estimates functionally relevant differences in root length distribution in maize and bean, but in agreement with our hypothesis, also found significant variation among phenotypes, replications, and environments. Systematic variation, attributable to coring location and phenotype, can be controlled for, but random variation arising from phenotypic plasticity, soil heterogeneity, and other unknown sources poses a significant challenge. Recognizing variation as dynamic, natural, and agroecologically important leads to a novel conceptualization framework, termed the rhizo canopy. A rhizo canopy is the result of dynamic interactions among an individual plant, its neighbors, and the pedosphere. It is analogous to a diverse forest canopy that has trees of variable height and is dynamic over time. Conceptualizing root length distribution as a spatiotemporally dynamic rhizo canopy may lead to a more nuanced perspective of soil exploration, useful to plant breeders and physiologists.

## Figures and Tables

**Table 1:** Quadratic discriminant analysis showing the misclassification rate of whole-plot RLD profiles for simulated deep, intermediate, and shallow phenotypes by different coring locations and combinations of locations. Only locations or combinations of coring locations with the lowest misclassification error rates are displayed. SE refers to standard error.

Coring location	Misclassification rate 3 maize phenotypes	Misclassification rate 3 bean phenotypes	Misclassification rate 2 maize phenotypes	Misclassification rate 2 bean phenotypes
1	0.31 (SE = 0.002)	0.51 (SE = 0.001)	0.07 (SE = 0.002)	0.32 (SE = 0.002)
2	0.37 (SE = 0.001)	0.48 (SE = 0.002)	0.11 (SE = 0.002)	0.26 (SE = 0.003)
3	0.47 (SE = 0.002)	0.36 (SE = 0.003)	0.25 (SE = 0.003)	0.1 (SE = 0.002)
6	0.24 (SE = 0.001)	0.57 (SE = 0.002)	0.13 (SE = 0.003)	0.43 (SE = 0.005)
6 and 1	0.23 (SE = 0.003)		0.07 (SE = 0.004)	
6 and 2	0.18 (SE = 0.001)		0.07 (SE = 0.003)	
6 and 3	0.25 (SE = 0.001)		0.1 (SE = 0.003)	
3 and 1		0.38 (SE = 0.002)		0.12 (SE = 0.003)
3 and 2		0.39 (SE = 0.003)		0.15 (SE = 0.004)
3 and 6		0.36 (SE = 0.003)		0.13 (SE = 0.003)

## Data Availability

The field and simulation data, model parameterization, R package to calculate Voronoi-adjusted root length distribution, and R scripts used to analyze data are available at Zenodo (https://doi.org/10.5281/zenodo.3952179).

## Abbreviations

ANOVA: One-way analysis of variance
D_50_: Depth where 50% of root length is found
D_95_: Depth where 95% of root length is found
P: Phosphorus
RSA: Root system architecture
RLD: Root length density
TOST: Two one-sided *T*-test.

## Additional Points

*Highlights*. Soil coring and trench excavations in the field and *in silico* soil coring of maize and bean were used to identify a soil coring location 5 cm from the plant base in the between-row direction, as most robust for estimating root length distribution. Variation of root length distribution in maize and bean is attributable to fixed factors such as coring location and random factors such as location in field and season. The rhizo canopy concept describes how a root length profile dynamically arises from the interactions among an individual plant, its neighbors, and the pedosphere.

## Supplementary Materials

Supplementary Materials Supplementary Figure 1: distribution of total root length of the three root system architecture (RSA) phenotypes generated by OpenSimRoot. Supplementary Figure 2: distribution of D95 for the different simulated maize phenotypes at the 6 different coring locations. Depth in cm is given on the *y* axis. Supplementary Figure 3: resampling of 2,000 simulation model fits testing the rate at which bean (top) and maize (bottom) RSA phenotypes are known to be different pass the 0.05 *p* value threshold for rejection of equal root length distribution. Soil core locations are given across the top as well as random core locations and a monolith (whole-plot). The rejection rate is on the *y* axis and number of replications on the *x* axis. The depth at which 50%, 80%, 90%, and 95% (*D*_50_, *D*_80_, *D*_90_, *D*_95_) of roots can be found is plotted for each location and species combination. Supplementary Figure 4: simulated RLD profile by phenotype (rows) and coring location (columns) with average whole-plot RLD profile overlaid in red on each panel. Supplementary Figure 5: panel A shows the Voronoi field-derived RLD profile for the 2 years of field data. Panel B shows the coefficient of variation by year and depth for the 2 years of field data, 2015 on the left side and 2016 on the right side. Supplementary Figure 6: root length by depth in 2015 (*x* axis) and 2016 (*y* axis) field trials by different locations (numbered) and species. The diagonal dashed line indicates 1 : 1 equivalence. Supplementary Figure 7: depth above which 50% (*D*_50_) of root length is found for simulated bean (top) and maize (bottom) phenotypes at the six different coring locations, a random core location, and a monolith. *Y* axis is depth and RSA phenotypes are on the *x* axis. Colors indicate number of sampling replicates. Supplementary Figure 8: depth above which 95% (*D*_95_) of root length is found for simulated bean (top) and maize (bottom) phenotypes at the six different coring locations, a random core location, and a monolith. *Y* axis is depth and RSA phenotypes are on the *x* axis. Colors indicate number of sampling replicates. Supplementary Figure 9: Heat map on the top left shows root length density (cm cm-3) and heat map on the bottom left shows a unitless visual score for fracturedness with 2 indicating more fractures. X's in the top left panel indicate plant stems. Photo of bean trench wall (right) following washing showing compacted zone in the center of the photo, cell P2. Additional supplementary materials available at, https://zenodo.org/record/3952179#.X1iMzWozZTY contain; data from field and simulation trials on root length distribution profiles of maize and common bean. Code used to conduct OpenSimRoot simulations. Code to calculate Voronoi area adjusted root length. Code to conduct QDA, and other analysis and plots presented in the manuscript. Supplementary Table 1: confusion matrix_two phenotypes. Supplementary material 2. Supplementary material 3.
