# Supplementary figures and images for: An Analysis of Soil Coring Strategies to Estimate Root Depth in Maize (Zea mays) and Common Bean (Phaseolus vulgaris)
Source: Plant Phenomics. 2020 Nov 8;2020:3252703. doi: 10.34133/2020/3252703 (PMC7706327; doi:10.34133/2020/3252703)

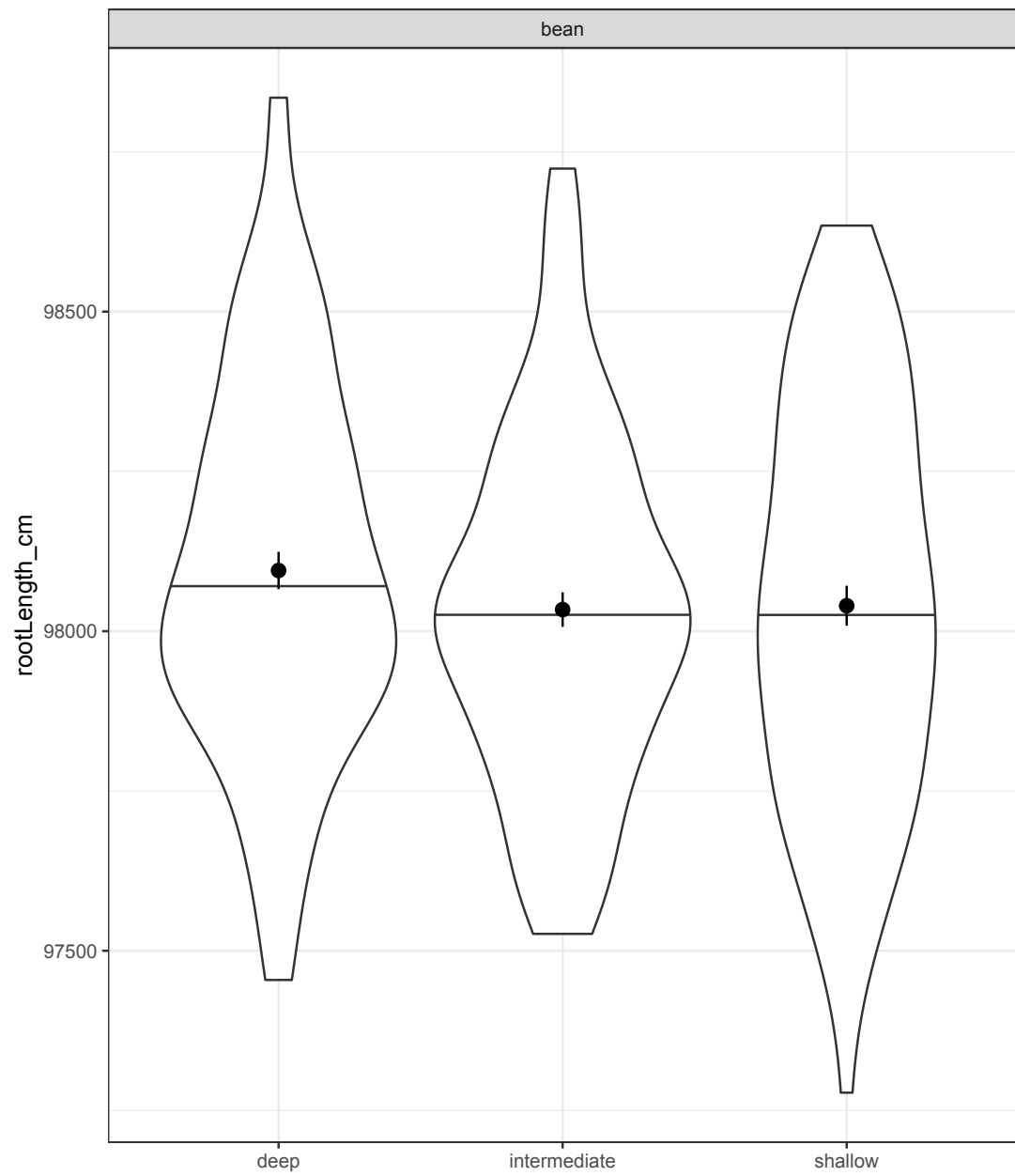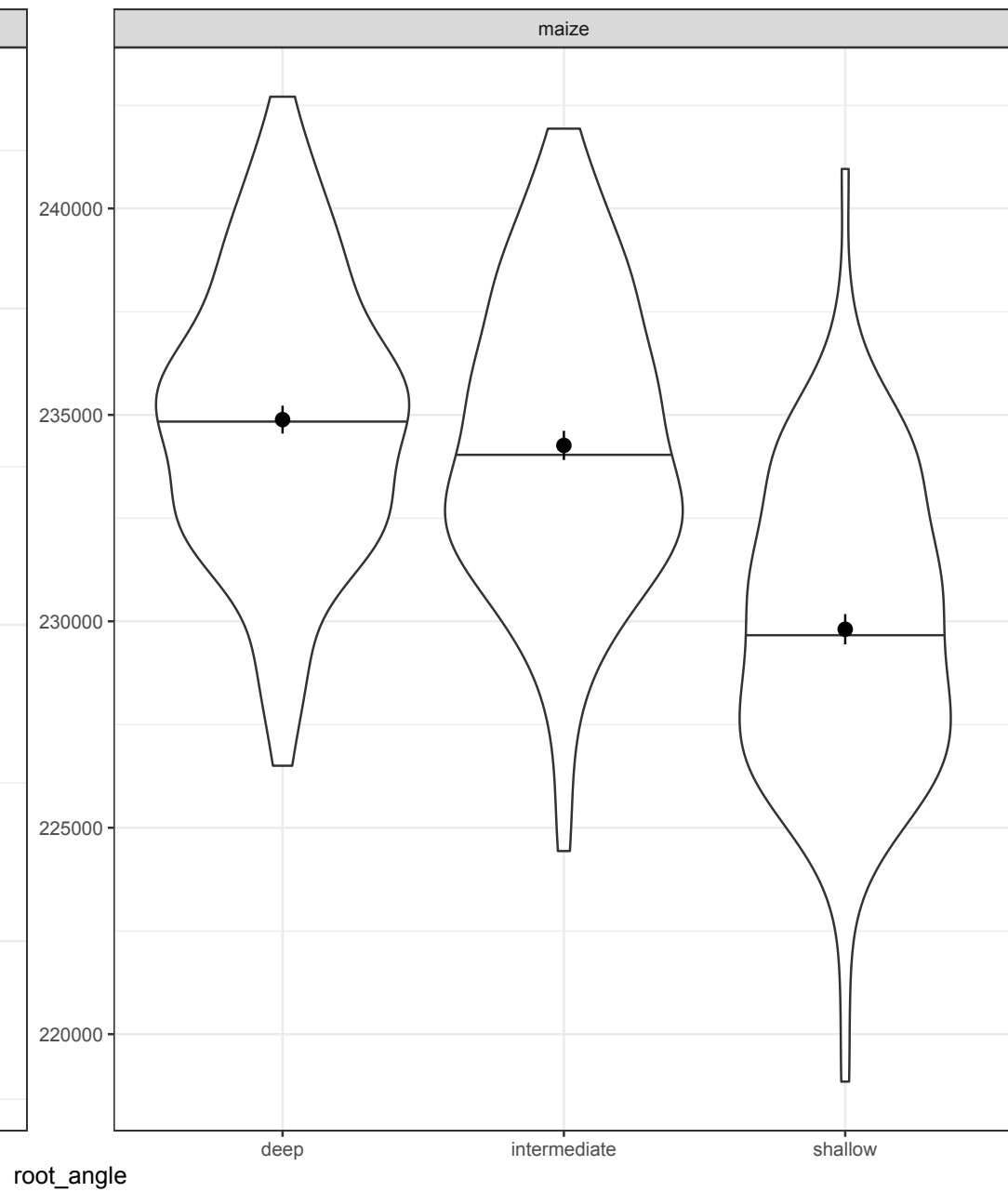

Supplement: Supplementary Materials — Supplementary Figure 1: distribution of total root length of the three root system architecture (RSA) phenotypes generated by OpenSimRoot. Supplementary Figure 2: distribution of D95 for the different simulated maize phenotypes at the 6 different coring locations. Depth in cm is given on the y axis. Supplementary Figure 3: resampling of 2,000 simulation model fits testing the rate at which bean (top) and maize (bottom) RSA phenotypes are known to be different pass the 0.05 p value threshold for rejection of equal root length distribution. Soil core locations are given across the top as well as random core locations and a monolith (whole-plot). The rejection rate is on the y axis and number of replications on the x axis. The depth at which 50%, 80%, 90%, and 95% (D50, D80, D90, D95) of roots can be found is plotted for each location and species combination. Supplementary Figure 4: simulated RLD profile by phenotype (rows) and coring location (columns) with average whole-plot RLD profile overlaid in red on each panel. Supplementary Figure 5: panel A shows the Voronoi field-derived RLD profile for the 2 years of field data. Panel B shows the coefficient of variation by year and depth for the 2 years of field data, 2015 on the left side and 2016 on the right side. Supplementary Figure 6: root length by depth in 2015 (x axis) and 2016 (y axis) field trials by different locations (numbered) and species. The diagonal dashed line indicates 1 : 1 equivalence. Supplementary Figure 7: depth above which 50% (D50) of root length is found for simulated bean (top) and maize (bottom) phenotypes at the six different coring locations, a random core location, and a monolith. Y axis is depth and RSA phenotypes are on the x axis. Colors indicate number of sampling replicates. Supplementary Figure 8: depth above which 95% (D95) of root length is found for simulated bean (top) and maize (bottom) phenotypes at the six different coring locations, a random core location, and a monolith. Y ax [file 3252703.f1.zip › 3252703.f1/Sup fig 1.pdf]

Phenotype ■ deep ■ intermediate ■ shallow

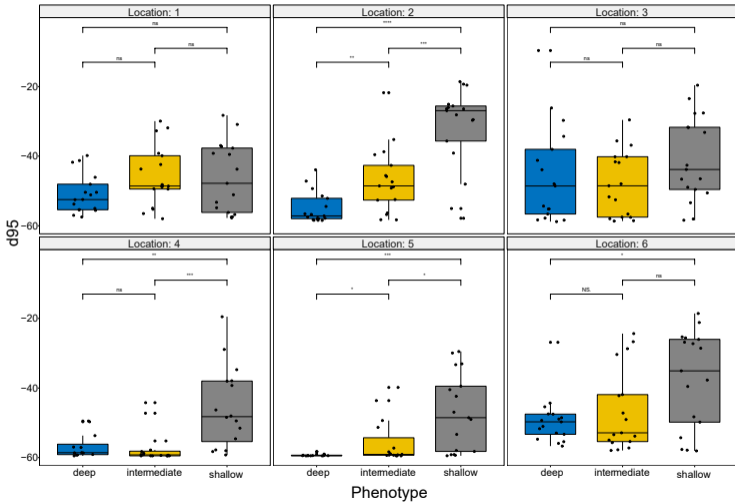

Supplement: Supplementary Materials — Supplementary Figure 1: distribution of total root length of the three root system architecture (RSA) phenotypes generated by OpenSimRoot. Supplementary Figure 2: distribution of D95 for the different simulated maize phenotypes at the 6 different coring locations. Depth in cm is given on the y axis. Supplementary Figure 3: resampling of 2,000 simulation model fits testing the rate at which bean (top) and maize (bottom) RSA phenotypes are known to be different pass the 0.05 p value threshold for rejection of equal root length distribution. Soil core locations are given across the top as well as random core locations and a monolith (whole-plot). The rejection rate is on the y axis and number of replications on the x axis. The depth at which 50%, 80%, 90%, and 95% (D50, D80, D90, D95) of roots can be found is plotted for each location and species combination. Supplementary Figure 4: simulated RLD profile by phenotype (rows) and coring location (columns) with average whole-plot RLD profile overlaid in red on each panel. Supplementary Figure 5: panel A shows the Voronoi field-derived RLD profile for the 2 years of field data. Panel B shows the coefficient of variation by year and depth for the 2 years of field data, 2015 on the left side and 2016 on the right side. Supplementary Figure 6: root length by depth in 2015 (x axis) and 2016 (y axis) field trials by different locations (numbered) and species. The diagonal dashed line indicates 1 : 1 equivalence. Supplementary Figure 7: depth above which 50% (D50) of root length is found for simulated bean (top) and maize (bottom) phenotypes at the six different coring locations, a random core location, and a monolith. Y axis is depth and RSA phenotypes are on the x axis. Colors indicate number of sampling replicates. Supplementary Figure 8: depth above which 95% (D95) of root length is found for simulated bean (top) and maize (bottom) phenotypes at the six different coring locations, a random core location, and a monolith. Y ax [file 3252703.f1.zip › 3252703.f1/Sup fig 2.pdf]

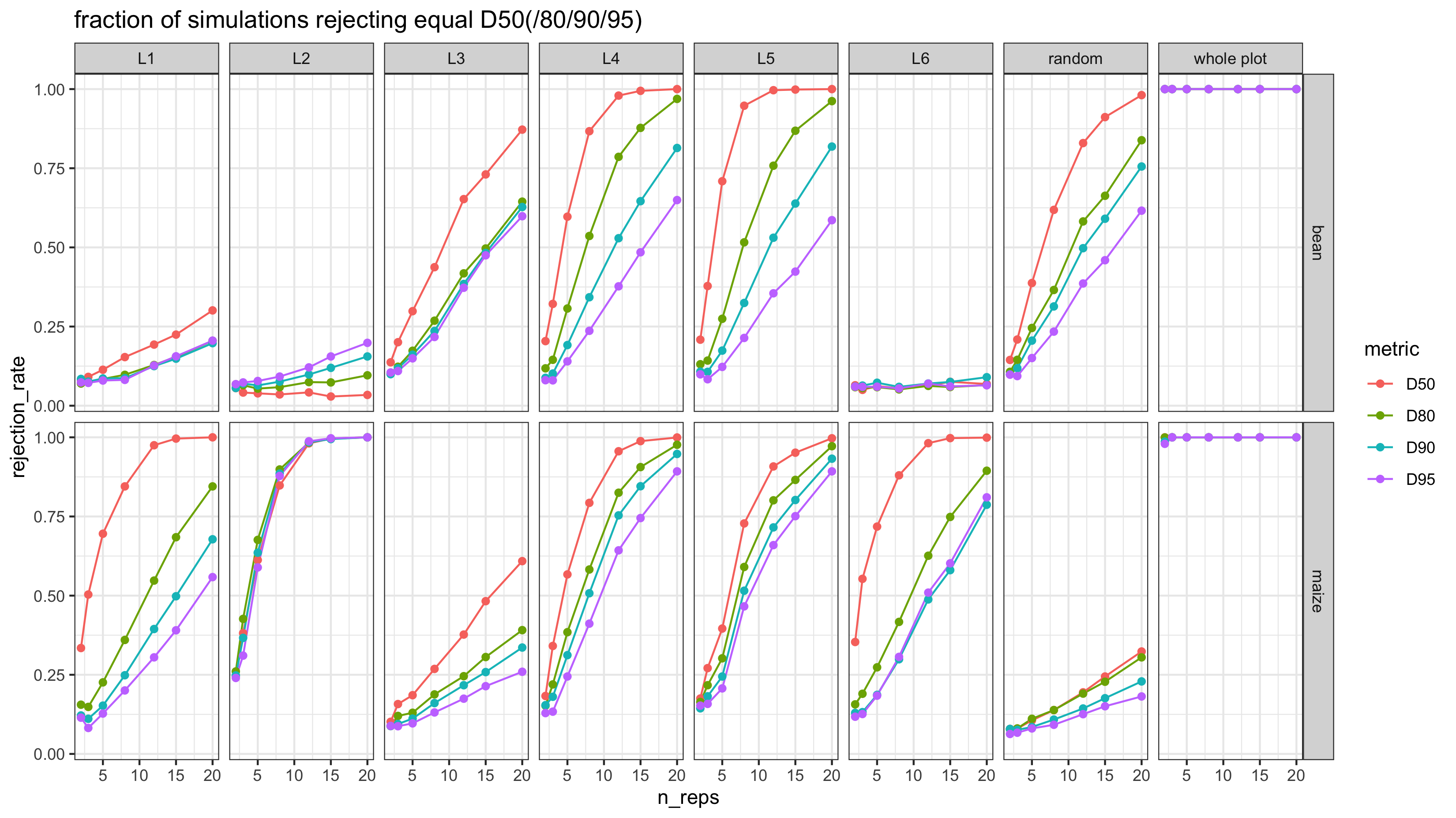

Supplement: Supplementary Materials — Supplementary Figure 1: distribution of total root length of the three root system architecture (RSA) phenotypes generated by OpenSimRoot. Supplementary Figure 2: distribution of D95 for the different simulated maize phenotypes at the 6 different coring locations. Depth in cm is given on the y axis. Supplementary Figure 3: resampling of 2,000 simulation model fits testing the rate at which bean (top) and maize (bottom) RSA phenotypes are known to be different pass the 0.05 p value threshold for rejection of equal root length distribution. Soil core locations are given across the top as well as random core locations and a monolith (whole-plot). The rejection rate is on the y axis and number of replications on the x axis. The depth at which 50%, 80%, 90%, and 95% (D50, D80, D90, D95) of roots can be found is plotted for each location and species combination. Supplementary Figure 4: simulated RLD profile by phenotype (rows) and coring location (columns) with average whole-plot RLD profile overlaid in red on each panel. Supplementary Figure 5: panel A shows the Voronoi field-derived RLD profile for the 2 years of field data. Panel B shows the coefficient of variation by year and depth for the 2 years of field data, 2015 on the left side and 2016 on the right side. Supplementary Figure 6: root length by depth in 2015 (x axis) and 2016 (y axis) field trials by different locations (numbered) and species. The diagonal dashed line indicates 1 : 1 equivalence. Supplementary Figure 7: depth above which 50% (D50) of root length is found for simulated bean (top) and maize (bottom) phenotypes at the six different coring locations, a random core location, and a monolith. Y axis is depth and RSA phenotypes are on the x axis. Colors indicate number of sampling replicates. Supplementary Figure 8: depth above which 95% (D95) of root length is found for simulated bean (top) and maize (bottom) phenotypes at the six different coring locations, a random core location, and a monolith. Y ax [file 3252703.f1.zip › 3252703.f1/Sup fig 3.png]

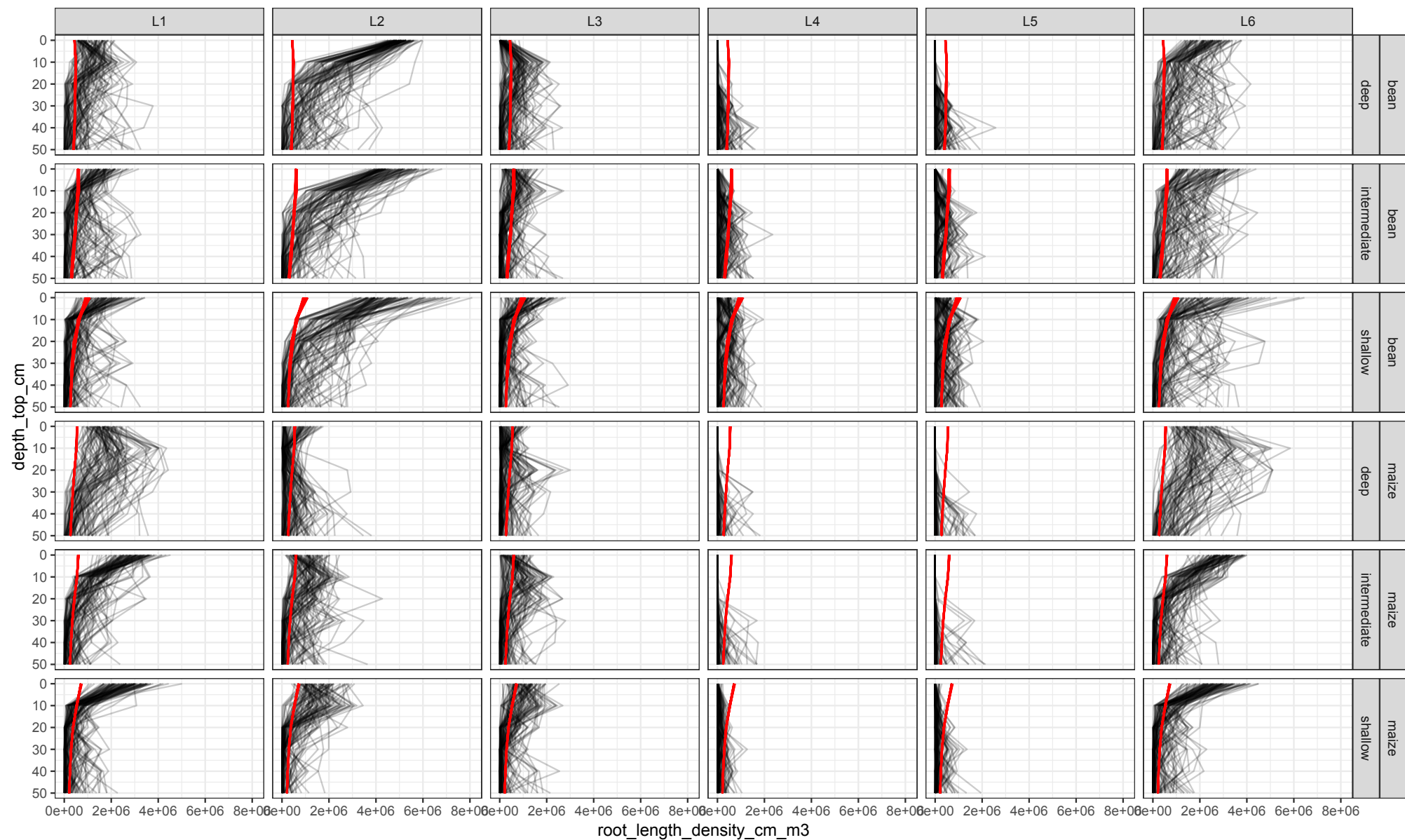

Supplement: Supplementary Materials — Supplementary Figure 1: distribution of total root length of the three root system architecture (RSA) phenotypes generated by OpenSimRoot. Supplementary Figure 2: distribution of D95 for the different simulated maize phenotypes at the 6 different coring locations. Depth in cm is given on the y axis. Supplementary Figure 3: resampling of 2,000 simulation model fits testing the rate at which bean (top) and maize (bottom) RSA phenotypes are known to be different pass the 0.05 p value threshold for rejection of equal root length distribution. Soil core locations are given across the top as well as random core locations and a monolith (whole-plot). The rejection rate is on the y axis and number of replications on the x axis. The depth at which 50%, 80%, 90%, and 95% (D50, D80, D90, D95) of roots can be found is plotted for each location and species combination. Supplementary Figure 4: simulated RLD profile by phenotype (rows) and coring location (columns) with average whole-plot RLD profile overlaid in red on each panel. Supplementary Figure 5: panel A shows the Voronoi field-derived RLD profile for the 2 years of field data. Panel B shows the coefficient of variation by year and depth for the 2 years of field data, 2015 on the left side and 2016 on the right side. Supplementary Figure 6: root length by depth in 2015 (x axis) and 2016 (y axis) field trials by different locations (numbered) and species. The diagonal dashed line indicates 1 : 1 equivalence. Supplementary Figure 7: depth above which 50% (D50) of root length is found for simulated bean (top) and maize (bottom) phenotypes at the six different coring locations, a random core location, and a monolith. Y axis is depth and RSA phenotypes are on the x axis. Colors indicate number of sampling replicates. Supplementary Figure 8: depth above which 95% (D95) of root length is found for simulated bean (top) and maize (bottom) phenotypes at the six different coring locations, a random core location, and a monolith. Y ax [file 3252703.f1.zip › 3252703.f1/Sup fig 4.pdf]

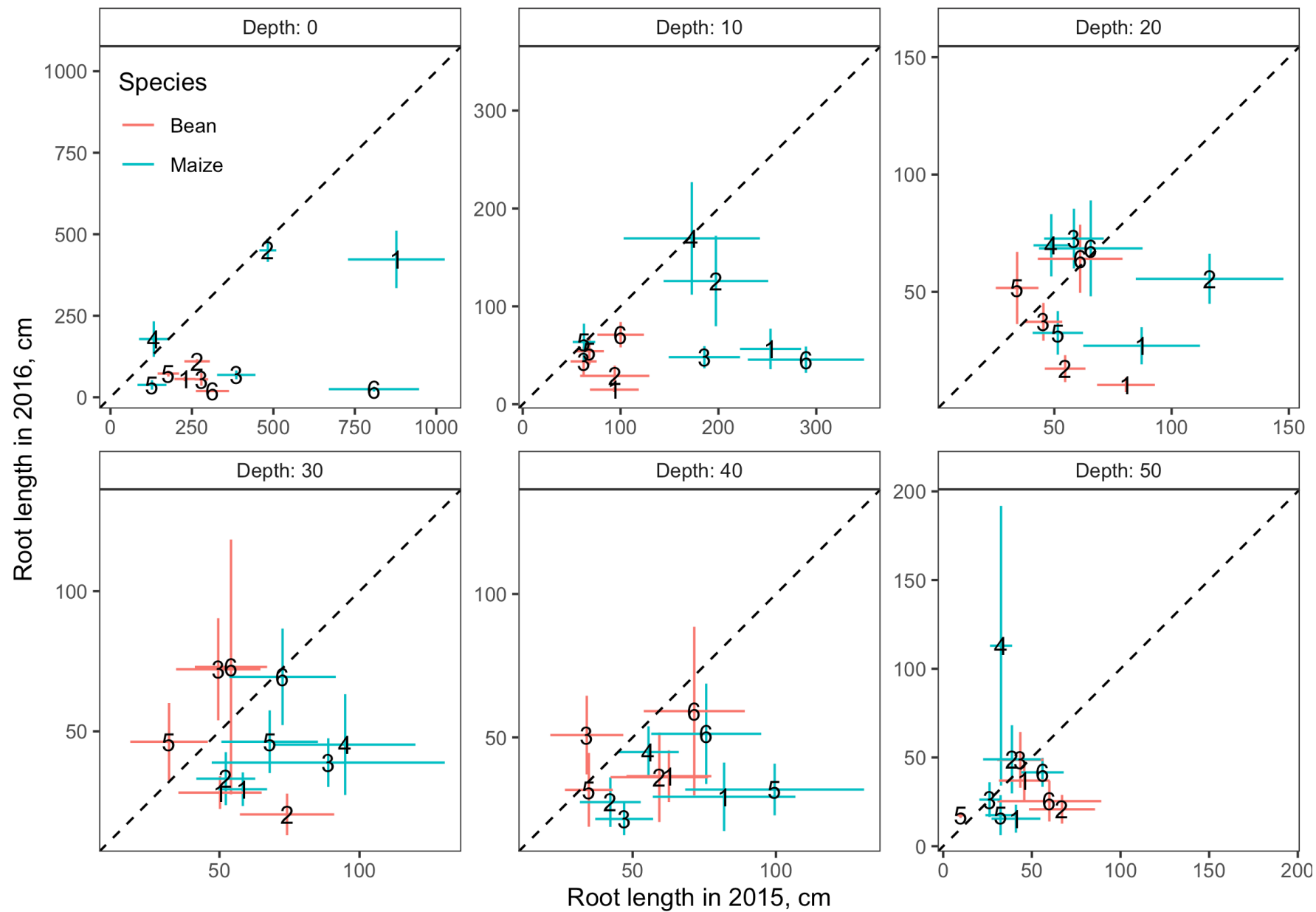

Supplement: Supplementary Materials — Supplementary Figure 1: distribution of total root length of the three root system architecture (RSA) phenotypes generated by OpenSimRoot. Supplementary Figure 2: distribution of D95 for the different simulated maize phenotypes at the 6 different coring locations. Depth in cm is given on the y axis. Supplementary Figure 3: resampling of 2,000 simulation model fits testing the rate at which bean (top) and maize (bottom) RSA phenotypes are known to be different pass the 0.05 p value threshold for rejection of equal root length distribution. Soil core locations are given across the top as well as random core locations and a monolith (whole-plot). The rejection rate is on the y axis and number of replications on the x axis. The depth at which 50%, 80%, 90%, and 95% (D50, D80, D90, D95) of roots can be found is plotted for each location and species combination. Supplementary Figure 4: simulated RLD profile by phenotype (rows) and coring location (columns) with average whole-plot RLD profile overlaid in red on each panel. Supplementary Figure 5: panel A shows the Voronoi field-derived RLD profile for the 2 years of field data. Panel B shows the coefficient of variation by year and depth for the 2 years of field data, 2015 on the left side and 2016 on the right side. Supplementary Figure 6: root length by depth in 2015 (x axis) and 2016 (y axis) field trials by different locations (numbered) and species. The diagonal dashed line indicates 1 : 1 equivalence. Supplementary Figure 7: depth above which 50% (D50) of root length is found for simulated bean (top) and maize (bottom) phenotypes at the six different coring locations, a random core location, and a monolith. Y axis is depth and RSA phenotypes are on the x axis. Colors indicate number of sampling replicates. Supplementary Figure 8: depth above which 95% (D95) of root length is found for simulated bean (top) and maize (bottom) phenotypes at the six different coring locations, a random core location, and a monolith. Y ax [file 3252703.f1.zip › 3252703.f1/Sup fig 6.pdf]

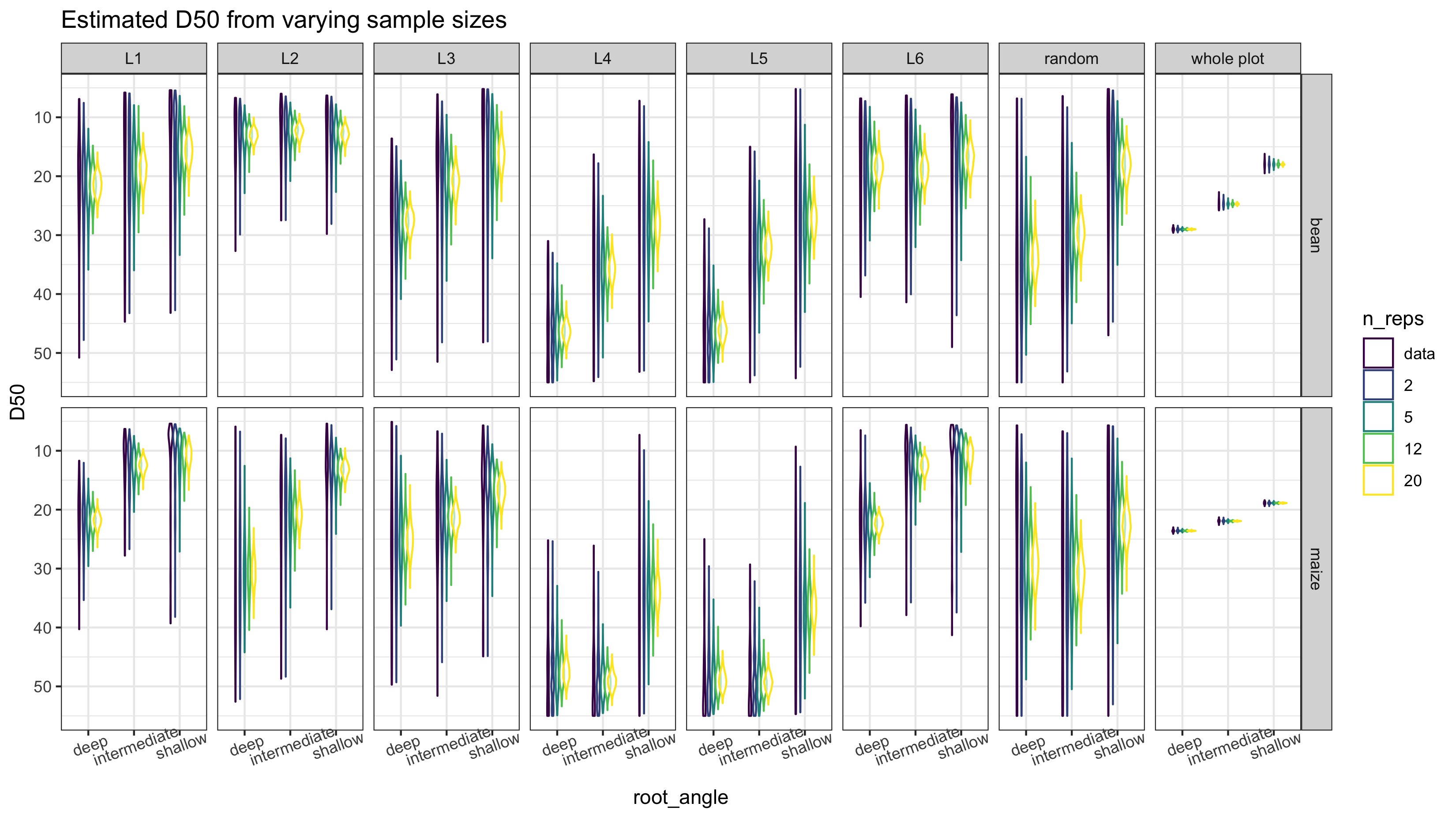

Supplement: Supplementary Materials — Supplementary Figure 1: distribution of total root length of the three root system architecture (RSA) phenotypes generated by OpenSimRoot. Supplementary Figure 2: distribution of D95 for the different simulated maize phenotypes at the 6 different coring locations. Depth in cm is given on the y axis. Supplementary Figure 3: resampling of 2,000 simulation model fits testing the rate at which bean (top) and maize (bottom) RSA phenotypes are known to be different pass the 0.05 p value threshold for rejection of equal root length distribution. Soil core locations are given across the top as well as random core locations and a monolith (whole-plot). The rejection rate is on the y axis and number of replications on the x axis. The depth at which 50%, 80%, 90%, and 95% (D50, D80, D90, D95) of roots can be found is plotted for each location and species combination. Supplementary Figure 4: simulated RLD profile by phenotype (rows) and coring location (columns) with average whole-plot RLD profile overlaid in red on each panel. Supplementary Figure 5: panel A shows the Voronoi field-derived RLD profile for the 2 years of field data. Panel B shows the coefficient of variation by year and depth for the 2 years of field data, 2015 on the left side and 2016 on the right side. Supplementary Figure 6: root length by depth in 2015 (x axis) and 2016 (y axis) field trials by different locations (numbered) and species. The diagonal dashed line indicates 1 : 1 equivalence. Supplementary Figure 7: depth above which 50% (D50) of root length is found for simulated bean (top) and maize (bottom) phenotypes at the six different coring locations, a random core location, and a monolith. Y axis is depth and RSA phenotypes are on the x axis. Colors indicate number of sampling replicates. Supplementary Figure 8: depth above which 95% (D95) of root length is found for simulated bean (top) and maize (bottom) phenotypes at the six different coring locations, a random core location, and a monolith. Y ax [file 3252703.f1.zip › 3252703.f1/Sup fig 7.png]

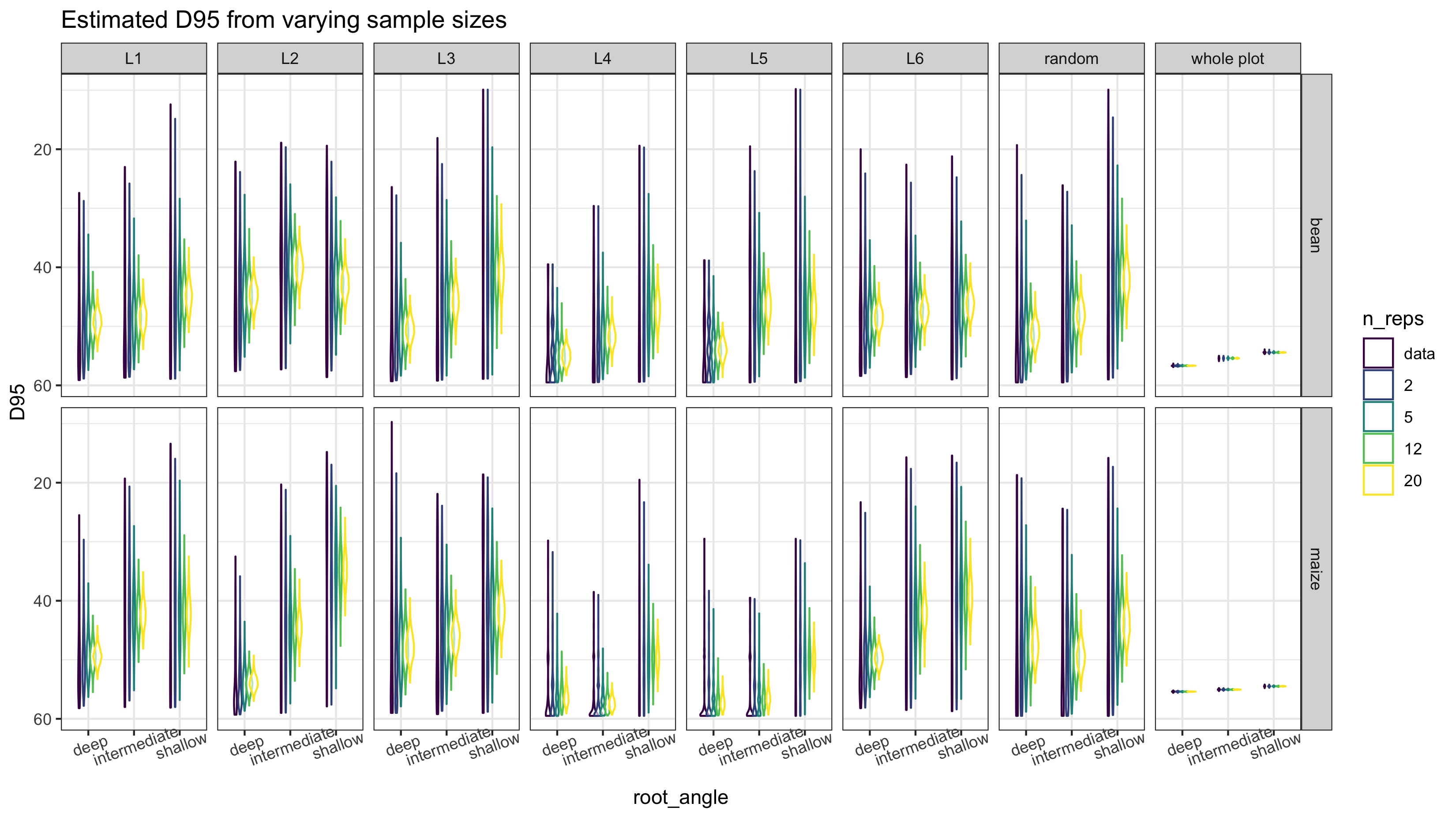

Supplement: Supplementary Materials — Supplementary Figure 1: distribution of total root length of the three root system architecture (RSA) phenotypes generated by OpenSimRoot. Supplementary Figure 2: distribution of D95 for the different simulated maize phenotypes at the 6 different coring locations. Depth in cm is given on the y axis. Supplementary Figure 3: resampling of 2,000 simulation model fits testing the rate at which bean (top) and maize (bottom) RSA phenotypes are known to be different pass the 0.05 p value threshold for rejection of equal root length distribution. Soil core locations are given across the top as well as random core locations and a monolith (whole-plot). The rejection rate is on the y axis and number of replications on the x axis. The depth at which 50%, 80%, 90%, and 95% (D50, D80, D90, D95) of roots can be found is plotted for each location and species combination. Supplementary Figure 4: simulated RLD profile by phenotype (rows) and coring location (columns) with average whole-plot RLD profile overlaid in red on each panel. Supplementary Figure 5: panel A shows the Voronoi field-derived RLD profile for the 2 years of field data. Panel B shows the coefficient of variation by year and depth for the 2 years of field data, 2015 on the left side and 2016 on the right side. Supplementary Figure 6: root length by depth in 2015 (x axis) and 2016 (y axis) field trials by different locations (numbered) and species. The diagonal dashed line indicates 1 : 1 equivalence. Supplementary Figure 7: depth above which 50% (D50) of root length is found for simulated bean (top) and maize (bottom) phenotypes at the six different coring locations, a random core location, and a monolith. Y axis is depth and RSA phenotypes are on the x axis. Colors indicate number of sampling replicates. Supplementary Figure 8: depth above which 95% (D95) of root length is found for simulated bean (top) and maize (bottom) phenotypes at the six different coring locations, a random core location, and a monolith. Y ax [file 3252703.f1.zip › 3252703.f1/Sup fig 8.png]

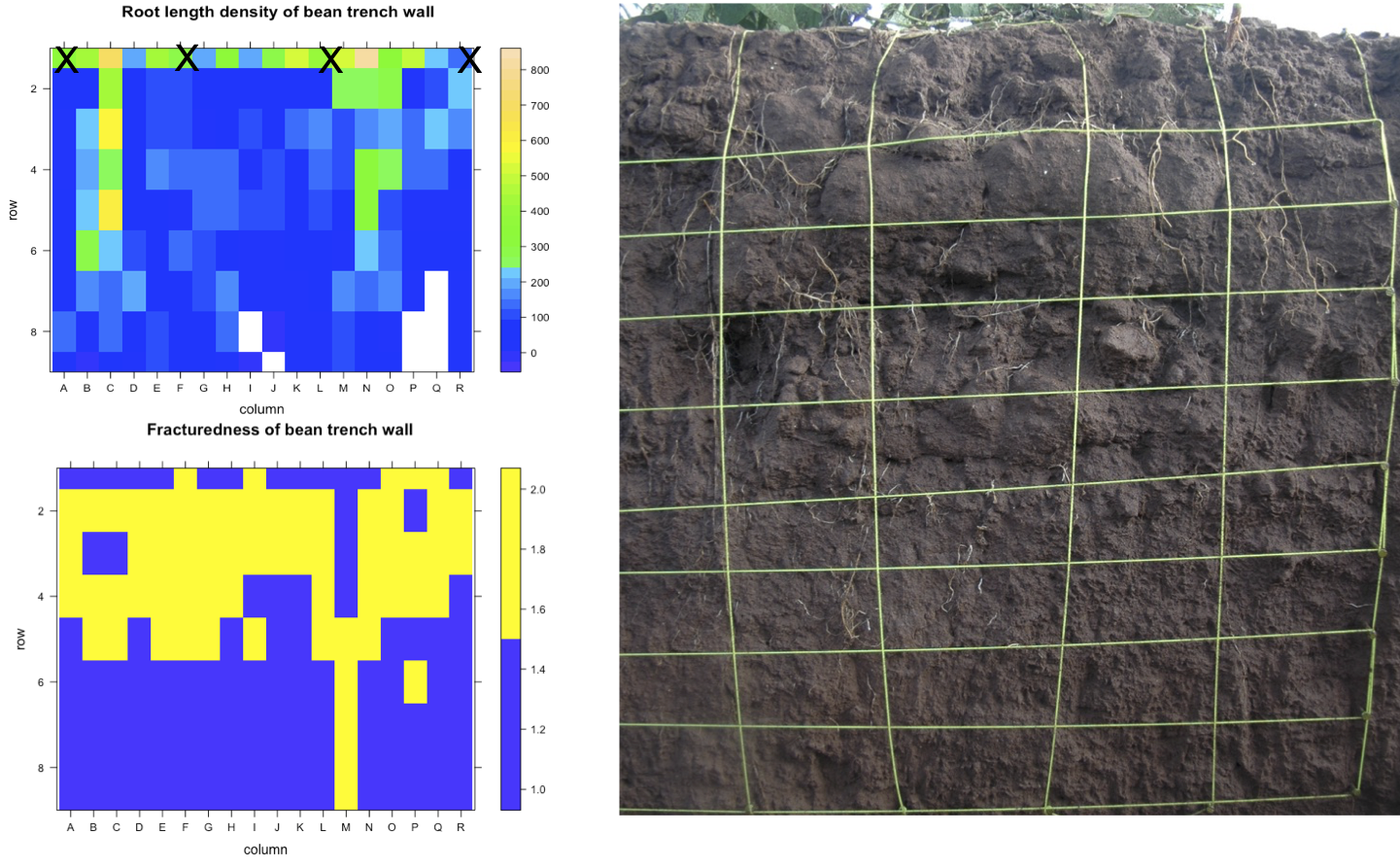

Supplement: Supplementary Materials — Supplementary Figure 1: distribution of total root length of the three root system architecture (RSA) phenotypes generated by OpenSimRoot. Supplementary Figure 2: distribution of D95 for the different simulated maize phenotypes at the 6 different coring locations. Depth in cm is given on the y axis. Supplementary Figure 3: resampling of 2,000 simulation model fits testing the rate at which bean (top) and maize (bottom) RSA phenotypes are known to be different pass the 0.05 p value threshold for rejection of equal root length distribution. Soil core locations are given across the top as well as random core locations and a monolith (whole-plot). The rejection rate is on the y axis and number of replications on the x axis. The depth at which 50%, 80%, 90%, and 95% (D50, D80, D90, D95) of roots can be found is plotted for each location and species combination. Supplementary Figure 4: simulated RLD profile by phenotype (rows) and coring location (columns) with average whole-plot RLD profile overlaid in red on each panel. Supplementary Figure 5: panel A shows the Voronoi field-derived RLD profile for the 2 years of field data. Panel B shows the coefficient of variation by year and depth for the 2 years of field data, 2015 on the left side and 2016 on the right side. Supplementary Figure 6: root length by depth in 2015 (x axis) and 2016 (y axis) field trials by different locations (numbered) and species. The diagonal dashed line indicates 1 : 1 equivalence. Supplementary Figure 7: depth above which 50% (D50) of root length is found for simulated bean (top) and maize (bottom) phenotypes at the six different coring locations, a random core location, and a monolith. Y axis is depth and RSA phenotypes are on the x axis. Colors indicate number of sampling replicates. Supplementary Figure 8: depth above which 95% (D95) of root length is found for simulated bean (top) and maize (bottom) phenotypes at the six different coring locations, a random core location, and a monolith. Y ax [file 3252703.f1.zip › 3252703.f1/Sup fig 9.png]
